# Application of Machine Learning in Modeling the Relationship between Catchment Attributes and Instream Water Quality in Data-Scarce Regions

**DOI:** 10.3390/toxics11120996

**Published:** 2023-12-07

**Authors:** Miljan Kovačević, Bahman Jabbarian Amiri, Silva Lozančić, Marijana Hadzima-Nyarko, Dorin Radu, Emmanuel Karlo Nyarko

**Affiliations:** 1 Faculty of Technical Sciences, University of Pristina, Knjaza Milosa 7, 38220 Kosovska Mitrovica, Serbia; 2 Faculty of Economics and Sociology, Department of Regional Economics and the Environment, 3/5 P.O.W. Street, 90-255 Lodz, Poland; bahman.amiri@uni.lodz.pl; 3 Faculty of Civil Engineering and Architecture Osijek, Josip Juraj Strossmayer University of Osijek, Vladimira Preloga 3, 31000 Osijek, Croatia; lozancic@gfos.hr (S.L.); mhadzima@gfos.hr (M.H.-N.); 4 Faculty of Civil Engineering, Department of Civil Engineering, Transilvania University of Brașov, 500152 Brașov, Romania; dorin.radu@unitbv.ro; 5 Faculty of Electrical Engineering, Computer Science and Information Technology Osijek, Josip Juraj Strossmayer University of Osijek, Kneza Trpimira 2B, 31000 Osijek, Croatia; karlo.nyarko@ferit.hr

**Keywords:** machine learning, water quality, land use, land cover, hydrologic soil groups, geological permeability

## Abstract

This research delves into the efficacy of machine learning models in predicting water quality parameters within a catchment area, focusing on unraveling the significance of individual input variables. In order to manage water quality, it is necessary to determine the relationship between the physical attributes of the catchment, such as geological permeability and hydrologic soil groups, and in-stream water quality parameters. Water quality data were acquired from the Iran Water Resource Management Company (WRMC) through monthly sampling. For statistical analysis, the study utilized 5-year means (1998–2002) of water quality data. A total of 88 final stations were included in the analysis. Using machine learning methods, the paper gives relations for 11 in-stream water quality parameters: Sodium Adsorption Ratio (SAR), Na^+^, Mg^2+^, Ca^2+^, SO_4_^2−^, Cl^−^, HCO^3−^, K^+^, pH, conductivity (EC), and Total Dissolved Solids (TDS). To comprehensively evaluate model performance, the study employs diverse metrics, including Pearson’s Linear Correlation Coefficient (R) and the mean absolute percentage error (MAPE). Notably, the Random Forest (RF) model emerges as the standout model across various water parameters. Integrating research outcomes enables targeted strategies for fostering environmental sustainability, contributing to the broader goal of cultivating resilient water ecosystems. As a practical pathway toward achieving a delicate balance between human activities and environmental preservation, this research actively contributes to sustainable water ecosystems.

## 1. Introduction

River water quality plays a crucial role in ensuring the sustainability and health of freshwater ecosystems. Traditional monitoring methods often have spatial and temporal coverage limitations, leading to difficulties in effectively assessing and managing water quality [1]. However, recent developments in machine learning techniques have indicated the potential to predict water quality accurately based on catchment characteristics [1,2]. One of the underlying reasons for the growing interest in applying machine learning techniques to predict river water quality is the ability to simultaneously consider a wide range of catchment characteristics. These characteristics include various features that affect water quality, including land use, soil properties, climate data, topography, and hydrological characteristics [3]. These variables interact in complex ways, and their associations may not easily be distinguished using prevalent analytical methods. In practice, for the modeling of water quality parameters, different mathematical models can be used that show satisfactory accuracy, as in papers [4,5]. A more comprehensive understanding of the factors influencing water quality can be achieved by applying machine learning algorithms, which can disclose hidden patterns and capture nonlinear relationships in large and diverse datasets [6].

The performance of supervised machine learning algorithms has been proved by recent studies in predicting river water quality [7]. These algorithms are trained on historical water quality data, in line with associating catchment characteristics, to explore the patterns and relationships between them [8]. Researchers have been able to develop accurate predictive models by applying algorithms such as decision trees, random forests, support vector machines, and neural networks [9,10,11]. Water quality parameters, such as nutrient concentrations, pollutant levels, and biological indicators, can then be applied by these models to make predictions using catchment characteristics [12]. Such predictions can help determine potential pollution points, prioritize management actions, and support decision-making processes for water resource management.

In addition to catchment characteristics, integrating remote sensing data has emerged as a valuable tool by which the accuracy of water quality predictions can be significantly enhanced [13,14], because the spatially explicit information about land cover/land use, vegetation, and surface characteristics can be provided by remote sensing techniques, which include satellite imagery and aerial photographs. Researchers can improve the predictive performance of machine learning models by combining remote sensing data with catchment characteristics [15]. For example, satellite data can provide insight into vegetation dynamics, land use/land cover changes, and the extent of impervious surfaces, which encompass urban and semi-urban areas, all of which can influence water quality. Integrating these additional spatial data sources into the machine learning models can result in more accurate and spatially explicit predictions, allowing a more comprehensive assessment of water quality dynamics across large river basins.

Furthermore, applying machine learning techniques to predict river water quality has led to collaborative efforts between researchers and stakeholders [16,17], which can be crucial to facilitate achieving the objectives of water resources management. These efforts aim to develop standardized frameworks and models that can be applied across different catchments and regions. Data sharing, methodologies, and the best practices can collectively improve the accuracy and reliability of predictive models that researchers develop. Collaborative initiatives also facilitate the identification of common challenges, such as data availability and quality issues, and foster the development of innovative solutions.

Advances in machine learning applications to predict river water quality underscore the growing importance of this field. Researchers are moving toward a more sustainable and informed water resource management by integrating advanced machine learning algorithms with catchment characteristics and remote sensing data. These predictive models can support policymakers, water resource managers, and environmental authorities in making evidence-based decisions, implementing targeted pollution control measures, and maintaining the ecological integrity of river ecosystems.

Integrating machine learning techniques with catchment characteristics gives researchers, water resource engineers, planners, and managers immense potential to predict river water quality. These models can provide accurate and timely information to support water resource management, pollution mitigation efforts, and the preservation of freshwater ecosystems by leveraging the power of advanced algorithms and incorporating diverse environmental data sources [18]. The advancements made in this field highlight the growing significance of machine learning in addressing the challenges associated with water quality prediction and paving the way for a more sustainable and informed management of our precious water resources.

Although machine learning applications in predicting river water quality based on catchment characteristics have shown promising findings, several gaps in this research field warrant further investigation. They include but are not limited to (1) data availability and quality, (2) the incorporation of temporal dynamics, (3) uncertainty estimation, (4) across-catchments transferability, (5) the integration of socio-economic factors, and (6) interpretability and transparency, which are briefly addressed as follows:

Data availability and quality, particularly historical water quality data and comprehensive catchment characteristics data remain challenges in many regions. Limited data may lead to biased or incomplete models, limiting the accuracy and the generality of predictions. Efforts should be made to improve data collection methods, establish standardized data protocols, and improve data sharing among researchers and stakeholders.

Current machine learning models often ignore the temporal dimension and assume static relationships between catchment characteristics and water quality parameters [19]. While integrating temporal dynamics into machine learning models could increase their predictive capacity and allow for more accurate short-term and long-term water quality forecasts, various temporal factors, such as seasonal variations, climate change, and short-term events such as precipitation events or pollution incidents, affect river water quality [20].

Machine learning models typically provide point predictions, but quantifying and communicating the uncertainties associated with these predictions is crucial for decision-making and risk assessment [21]. Developing methods to quantify and propagate uncertainties through the modeling process, considering the sources of uncertainty such as data quality, model structure, and parameter estimation, would enhance the reliability and applicability of predictive models.

The use of deep learning models is considered for modeling changes in water reservoirs. The methodology of Long Short-Term Memory (LSTM) networks was applied in the work, and a number of criteria including the Coefficient of Determination (R^2^), Root Mean Square Error (RMSE), mean absolute percentage error (MAPE), Mean Absolute Deviation (MAD), and Nash–Sutcliffe Efficiency (NSE) were used to assess the accuracy. Satisfactory accuracy of the model was achieved on a series of samples that covered the period from 2003 to 2025 for five basins in Saudi Arabia [22].

Research regarding the application of artificial intelligence techniques—artificial neural networks (ANN), a group method of data handling (GMDH), and support vector machines (SVM)—for predicting water quality components in Tireh River, southwest Iran showed that the application of ANN and SVM models, using tansig and RBF functions, respectively, showed satisfactory performance. The database included samples collected over a period of 55 years [23].

In addition, models developed for a particular catchment cannot be applied directly to another due to variations in catchment characteristics, land use/cover, soil, geology, and climatic conditions. The development of transferable models that can account for specific variations in catchment while taking general patterns would be valuable for the management of water resources on a larger scale [24]. On the other hand, capturing the characteristics related to human activities, such as agricultural practices, urbanization, and industrial activities, has a significant impact on water quality. Incorporating socio-economic factors into machine learning models can improve their predictive power and enable more comprehensive water quality assessments. However, the integration of socio-economic data and understanding the complex interactions between human activity and water quality present challenges that must be addressed.

It should be noted that the need for greater ambiguity and transparency in machine learning models can limit the adoption and acceptance of these models by policymakers and stakeholders [25]. The development of logical machine learning techniques that provide insights into the model decision-making process and highlight the most influential catch characteristics would improve the reliability and usability of predictive models.

Addressing these gaps requires interdisciplinary collaborations among hydrologists, ecologists, data scientists, policymakers, and other relevant stakeholders. Furthermore, focusing on data-driven approaches, data-sharing initiatives, and advances in computational methods will be critical to advancing the field and harnessing the full potential of machine learning in predicting river water quality based on catchment characteristics. In the study, we intend to investigate how we can address spatial variations in the characteristics of the catchment to explain river water quality using machine learning techniques.

This paper explores the relationship between catchment attributes and in-stream water quality parameters using machine learning methods. It evaluates model accuracy with RMSE, MAE, R, and MAPE, identifies optimal models for 11 parameters, and determines significant influencing variables.

The predictive models developed for each water parameter demonstrate strong performance in most cases. The significant variables identified provide insights into the key factors influencing water quality in the studied catchment. This research, therefore, serves as a catalyst for fostering a nuanced and effective approach to water resource management, underpinned by the empirical foundation laid by the predictive models and the discerned influential variables. As a result, the integration of these findings into decision-making processes holds the potential to optimize resource allocation, mitigate environmental impacts, and ultimately contribute to the overarching goal of achieving sustainable and resilient water ecosystems. The study highlights the potential of artificial intelligence for quick and accurate water quality assessment, tailored to watershed attributes.

## 2. Materials and Methods

To establish relationships between the catchment attributes and water quality parameters, machine learning methods were employed. Multiple algorithms, such as regression trees, TreeBagger, Random Forests, and Gaussian process regression (GPR) models, were applied to construct predictive models for each water quality parameter.

### 2.1. Regression Tree Models

**Figure 1:**
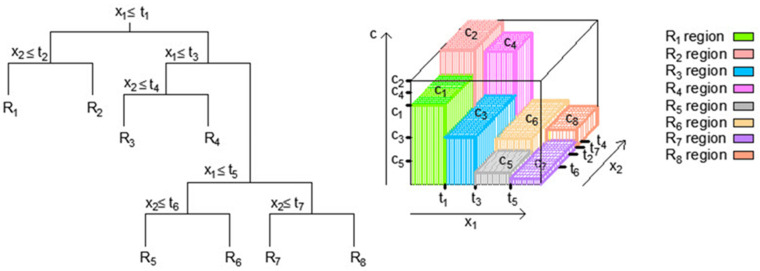
The partitioning of an input space into distinct regions and the representation of a 3D regression surface within a regression tree [26].

The fundamental concept behind regression trees is to partition the input space into distinct regions and assign predictive values to these regions. This segmentation enables the model to make predictions based on the most relevant conditions and characteristics of the data. A regression tree (RT) is a simple and comprehensible machine learning model applicable to both regression and classification problems. It follows a tree-like structure composed of nodes and branches (Figure 1).

Each node corresponds to a specific condition related to the input data, and this condition is evaluated at each node as the data progresses through the tree.

To predict an outcome for a given input, the starting point is the root node of the tree (Figure 1). Here, the initial condition associated with the input feature(s) is considered. Depending on whether this condition is deemed true or false, the branches are followed to reach the next node. This process is repeated recursively until a leaf node is arrived at. At the leaf node, a value is found, which serves as the predicted result for the input instance. For regression tasks, this value is typically a numeric prediction.

As the tree is traversed, the input space undergoes changes. Initially, all instances are part of a single set represented by the root node. However, as the algorithm progresses, the input space is gradually divided into smaller subsets. These divisions are based on conditions that help in tailoring predictions to different regions within the input space.

The process of constructing regression trees involves determining the optimal split variable (“j”) and split point (“s”) to partition the input space effectively. These variables are chosen by minimizing a specific expression (Equation (1)) that considers all input features. The goal is to minimize the sum of squared differences between observed values and predicted values in resulting regions [27,28,29].

(1) $$\underset{j,s}{\min}\left[\underset{c_{1}}{\min}\sum_{x_{i}\in R_{1}(j,s)}{\left(y_{i}-c_{1}\right)}^{2}+\underset{c_{2}}{\min}\sum_{x_{i}\in R_{2}(j,s)}{\left(y_{i}-c_{2}\right)}^{2}\right]$$

Once “j” and “s” are identified, the tree-building process continues by iteratively dividing regions. This process is referred to as a “greedy approach” because it prioritizes local optimality at each step. The binary recursive segmentation approach divides the input space into non-overlapping regions characterized by their mean values.

The depth of a regression tree serves as a critical factor in preventing overfitting (too much detail) or underfitting (too simplistic).

### 2.2. Ensembles of Regression Trees: Bagging, Random Forest, and Boosted Trees

Bagging is another ensemble method that involves creating multiple subsets of the training dataset through random sampling with replacement (bootstrap samples).

The process begins with the creation of multiple bootstrap samples from the original dataset. Bootstrap sampling involves randomly selecting data points from the dataset with replacement. This means that the same data point can be selected multiple times, while others may not be selected at all. In this way, subsets of the same size as the original data set are formed and are used to train the model.

**Figure 2:**
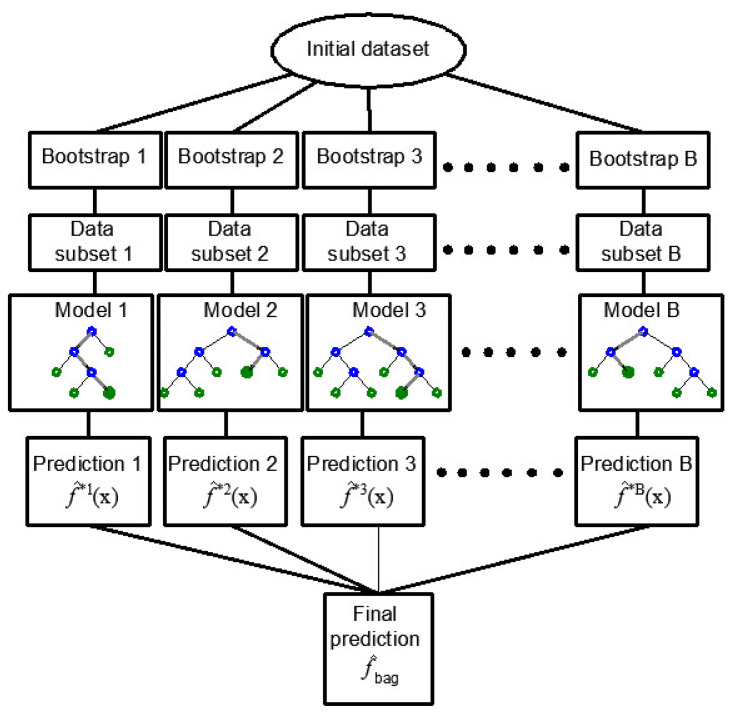
Creating regression tree ensembles using the bagging approach [30].

Each subset is used to train a separate regression tree model, and their predictions are aggregated to make the final prediction (Figure 2). Bagging helps reduce variance by averaging predictions from multiple models, making it particularly effective when the base models are unstable or prone to overfitting [27,28,29].

In bagging, multiple training sets are generated by repeatedly selecting samples from the original dataset, and this process involves sampling with replacement. This technique is utilized to create diverse subsets of data. The primary goal is to reduce the variance in the model’s predictions by aggregating the results from these different subsets. Consequently, each subset contributes to the final prediction, and the averaging of multiple models enhances the model’s robustness and predictive accuracy.

Random forests, a variant of bagging, stand out by introducing diversity among the constituent models within the ensemble. This diversity is achieved through the creation of multiple regression trees, each trained on a distinct bootstrap sample from the data. Moreover, before making decisions at each split within these trees, only a randomly selected subset of available features is considered. This approach helps in decorrelating the individual trees within the ensemble, thereby further reducing variance. The ensemble’s final prediction is generated by aggregating the predictions from these decorrelated trees, resulting in a robust and high-performing model [26,27,28,29].

**Figure 3:**
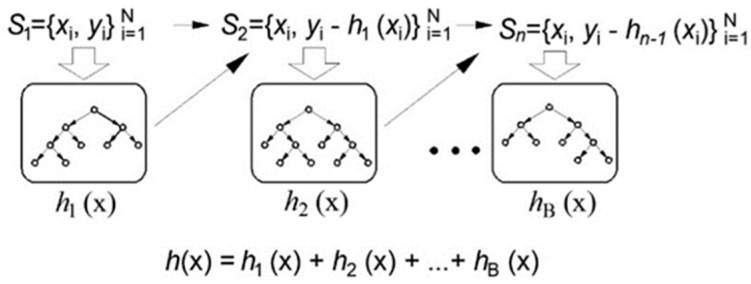
The application of gradient boosting within regression tree ensembles [26].

The boosting tree method is a sequential training method, and within this paradigm, gradient boosting stands out as a widely employed technique for enhancing overall model performance (Figure 3). In gradient boosting, submodels are introduced iteratively, with each new model selected based on its capacity to effectively estimate the residuals or errors of the preceding model in the sequence. The distinctive feature of gradient boosting lies in its commitment to minimizing these residuals during the iterative process.

By focusing on minimizing residuals, gradient boosting ensures that each new submodel added to the ensemble is adept at correcting the errors left by its predecessors. This emphasis on addressing the shortcomings of prior models leads to the creation of a robust and adaptive ensemble model. The iterative nature of gradient boosting allows it to systematically refine its predictions, making the final ensemble proficient in capturing intricate patterns and nuances within the data. The result is a powerful model capable of delivering highly accurate predictions by continuously learning and adapting to the complexities present in the dataset.

The fundamental concept is rooted in gradient-based optimization techniques, which involve refining the current solution to an optimization problem by incorporating a vector that is directly linked to the negative gradient of the function under minimization, as referenced in previous works [31,32,33]. This approach is logical because a negative gradient signifies the direction in which the function decreases. When it is applied a quadratic error function, each subsequent model aims to correct the discrepancies left by its predecessors, essentially reinforcing and improving the model with a focus on the residual errors from earlier stages.

In the context of gradient-boosting trees, the learning rate is a crucial hyperparameter that controls the contribution of each tree in the ensemble to the final prediction. It is often denoted as “lambda” (λ). The learning rate determines how quickly or slowly the model adapts to the errors from the previous trees during the boosting process. A lower learning rate means that the model adjusts more gradually and may require a larger number of trees to achieve the same level of accuracy, while a larger learning rate leads to faster adaptation but may risk overfitting with too few trees.

BT models are significantly more complex regarding computational complexity because they are trained sequentially compared to TR and RF models that can be trained in parallel.

### 2.3. Gaussian Process Regression (GPR)

Gaussian processes provide a probabilistic framework for modeling functions, capturing uncertainties, and making predictions in regression tasks. The choice of covariance functions and hyperparameters allows for flexibility in modeling relationships among variables [34].

Gaussian process modeling involves estimating an unknown function f(∙) in nonlinear regression problems. It assumes that this function follows a Gaussian distribution characterized by a mean function μ(∙) and a covariance function k(∙,∙). The covariance matrix K is a fundamental component of GPR and is determined by the kernel function (k).

The kernel function (k) plays a pivotal role in capturing the relationships between input data points (x and x′). This function is essential for quantifying the covariance or similarity between random values f(x) and f(x′). One of the most widely used kernels is defined by the following expression:

(2) $$k\left(xx^{\prime }\right)=\sigma^{2}exp\left(-\frac{{\left(x-x^{\prime }\right)}^{2}}{2l^{2}}\right)$$

In this expression, several elements are critical:

$\sigma^{2}$ represents the signal variance, a model parameter that quantifies the overall variability or magnitude of the function values.

The exponential function “exp” is used to model the similarity between x and x′. It decreases as the difference between x and x′ increases, capturing the idea that values close to each other are more strongly correlated.

The parameter *l*, known as the length scale, is another model parameter. It controls the smoothness and spatial extent of the correlation. A smaller *l* results in more rapid changes in the function, while a larger *l* leads to smoother variations.

The observations in a dataset $y=\left\{y_{1},\ldots ,y_{n}\right\}$ can be viewed as a sample from a multivariate Gaussian distribution.

(3) $${\left(y_{1},\ldots ,y_{n}\right)}^{T}\sim N(\mu ,\;Κ),$$

Gaussian processes are employed to model the relationship between input variables x and target variables y, considering the presence of additive noise $\varepsilon \sim Ν(0,\;\sigma^{2})$. The goal is to estimate the unknown function f(∙). The observations y are treated as a sample from a multivariate Gaussian distribution with mean vector μ and covariance matrix Κ. This distribution captures the relationships among the data points. The conditional distribution of a test point’s response value $y^{*}$, given the observed data $y={\left(y_{1},\ldots ,y_{n}\right)}^{T}$, is represented as $N({\hat{y}}^{*},{{\hat{\sigma }}^{*}}^{2})$ with the following:

(4) $${\hat{y}}^{*}=\mu \left(x^{*}\right)+{\;K}^{*T}{\;K}^{-1}\left(y-\mu \right),$$

(5) $${{\hat{\sigma }}^{*}}^{2}={\;K}^{**}+\sigma^{2}-{\;K}^{*T}{\;K}^{-1}K^{*}.$$

In traditional GPR, a single length-scale parameter (*l*) and signal variance ($\sigma^{2}$) are used for all input dimensions. In contrast, the Automatic Relevance Determination (ARD) approach employs a separate length-scale parameter ($l_{i}$) for each input dimension, where ‘i’ represents a specific dimension. This means that for a dataset with ‘m’ input dimensions, you have ‘m’ individual length-scale parameters [34].

The key advantage of ARD is that it automatically determines the relevance of each input dimension in the modeling process. By allowing each dimension to have its own length scale parameter, the model can assign different degrees of importance to each dimension. This means that the model can adapt and focus more on dimensions that are more relevant to the target variable and be less influenced by less relevant dimensions.

GPR involves matrix operations, and the computational complexity can become an issue for large datasets. Techniques such as sparse approximations or using specialized kernels can be employed to address these computational challenges. GPR is frequently used in Bayesian optimization problems where the goal is to optimize an unknown objective function that is expensive to evaluate.

## 3. Case Study of the Caspian Sea Basin

**Figure 4:**
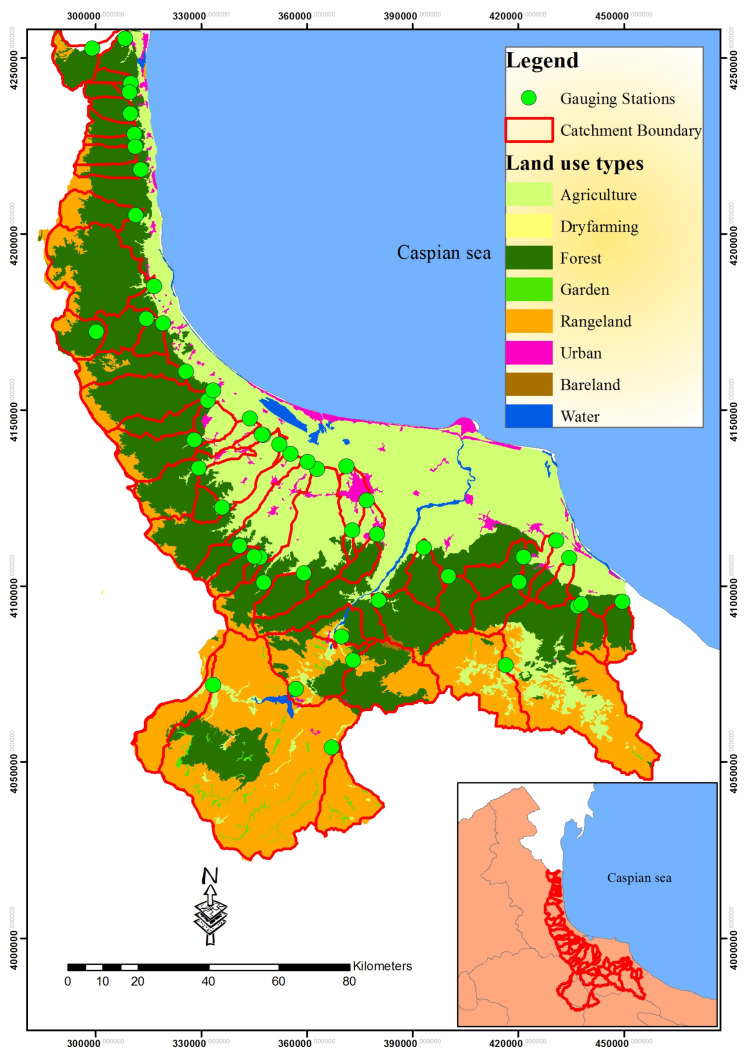
The study region and catchment areas situated within the southern Caspian Sea basin.

This study took place in the Caspian Sea catchment area (Figure 4) in Northern Iran, covering approximately 618 m^2^ with coordinates ranging from 49°48′ to 54°41′ longitude and from 35°36′ to 37°19′ latitude (Figure 4).

The majority of this area, approximately 65.10%, is forested, while the rest consists of rangelands (24.41%), agricultural land (9.41%), urban areas (0.88%), water bodies (0.0126%), and bare land (0.186%) [35].

Initially, 108 water quality monitoring stations scattered across the southern basin of the Caspian Sea were selected for analysis (Figure 4). To define the upstream catchment boundaries, digital elevation models (DEMs) with a resolution of 30 m by 30 m from the USGS database were used, with boundary refinement achieved through a user digitizing technique. Macro-sized catchments, those exceeding 1000 square kilometers, totaling 18 catchments, were excluded from the modeling process due to their significant impact on hydrological dynamics.

Water quality data, including parameters like SAR, Na^+^, Mg^2+^, Ca^2+^, SO_4_^2−^, Cl^−^, HCO^3−^, K^+^, pH, EC, and TDS, were obtained from the Iran Water Resource Management Company (WRMC) through monthly sampling. Collection adhered to the WRMC Guidelines for Surface Water Quality Monitoring (2009) and EPA-841-B-97-003 standards [36]. For statistical analysis, the 5-year means (1998–2002) of water quality data were calculated. After scrutinizing for normality and identifying outliers, 88 final stations were used in the study. The geographic scope of the study area is illustrated in Figure 4.

A land cover dataset was created using a 2002 digital land cover map (Scale 1:250,000) from the Forest, Ranges, and Watershed Management Organization of Iran. The original land cover categories were consolidated into six classes: bare land, water bodies, urban areas, agriculture, rangeland, and forests, following [37] land use and land cover classification systems. Furthermore, digital geological and soil feature maps (1:250,000 scale) were obtained from the Geological Survey of Iran (www.gsi.ir, accessed on 24 April 2021). Detailed information about the characteristics of the catchments and their statistical attributes can be found in Table 1 and Table 2.

In this study, hydrologic soil groups and geological permeability classes were developed and applied in conjunction with land use/land cover types within the modeling process. Hydrologic soil groups are influenced by runoff potential and can be used to determine runoff curve numbers. They consist of four classes (A, B, C, and D), with A having the highest runoff potential and D the lowest. Notably, soil profiles can undergo significant alterations due to changes in land use/land cover. In such cases, the soil textures of the new surface soil can be employed to determine the hydrologic soil groups as described in Table 1 [38]. Furthermore, the study incorporates the application of geological permeability attributes related to catchments, with the development of three geological permeability classes: Low, Medium, and High. These classes are associated with various characteristics of geological formations, such as effective porosity, cavity type and size, their connectivity, rock density, pressure gradient, and fluid properties like viscosity.

The range and statistical properties of training and test data play a fundamental role in the development and evaluation of machine learning models. They impact the model’s generalization, robustness, fairness, and ability to perform effectively in diverse real-world scenarios. Statistical properties of input and output data are given in Table 1 and Table 2.

The machine learning methods used in this paper were assessed using five-fold cross-validation. In this approach, the dataset was randomly divided into five subsets, with four of them dedicated to training the model and the remaining subset utilized for model validation (testing). This five-fold cross-validation process was repeated five times, ensuring that each subset was used exactly once for validation. Subsequently, the results from these five repetitions were averaged to produce a single estimation.

All models were trained and tested under identical conditions, ensuring a fair and consistent evaluation of their performance. This practice is essential in machine learning to provide a level playing field for comparing different algorithms and models.

When machine learning models are trained and tested under equal conditions, it means that they are exposed to the same datasets, preprocessing steps, and evaluation metrics.

The quality of the model was assessed using several evaluation and performance measures, which include RMSE, MAE, Pearson’s Linear Correlation Coefficient (R), and MAPE.

The RMSE criterion, expressed in the same units as the target values, serves as a measure of the model’s general accuracy. It is calculated as the square root of the average squared differences between the actual values ($d_{k}$) and the model’s predictions ($o_{k}$) across the training samples (N).

(6) $$RMSE=\sqrt{\frac{1}{N}\sum_{k=1}^{N}{\left(d_{k}-o_{k\;}\right)}^{2},}$$

The MAE criterion represents the mean absolute error of the model, emphasizing the absolute accuracy. It calculates the average absolute differences between the actual values and the model’s predictions.

(7) $$MAE=\frac{1}{N}\sum_{k=1}^{N}\left|d_{k}-o_{k\;}\right|.$$

Pearson’s Linear Correlation Coefficient (R) provides a relative measure of accuracy assessment. It considers the correlation between the actual values ($d_{k}$) and the model’s predictions ($o_{k}$) relative to their respective means ($\bar{d}$ and $\bar{o}$). Values of R greater than 0.75 indicate a strong correlation between the variables.

(8) $$R=\frac{\sum_{k=1}^{N}(d_{k}-\bar{d})(o_{k}-\bar{o})}{\sqrt{\left[\sum_{k=1}^{N}{\left(d_{k}-\bar{d}\right)}^{2}{\left(o_{k}-\bar{o}\right)}^{2}\right]}}.$$

The MAPE is a relative criterion that evaluates accuracy by calculating the average percentage differences between the actual values and the model’s predictions.

(9) $$MAPE=\frac{100}{N}\sum_{k=1}^{N}\left|\frac{d_{k}-o_{k\;}}{d_{k}}\right|.$$

This research deals with a limited dataset, and in this case, there is a higher risk of overfitting, where a model performs well on the training data but needs to generalize to new, unseen data. Five-fold cross-validation helps mitigate overfitting by partitioning the dataset into five subsets, using four for training and one for testing in each iteration. This process allows for a more robust evaluation of the model’s performance.

Five-fold cross-validation efficiently utilizes the available data by rotating through different subsets for training and testing, ensuring that each data point contributes to training and evaluation.

**Figure 5:**
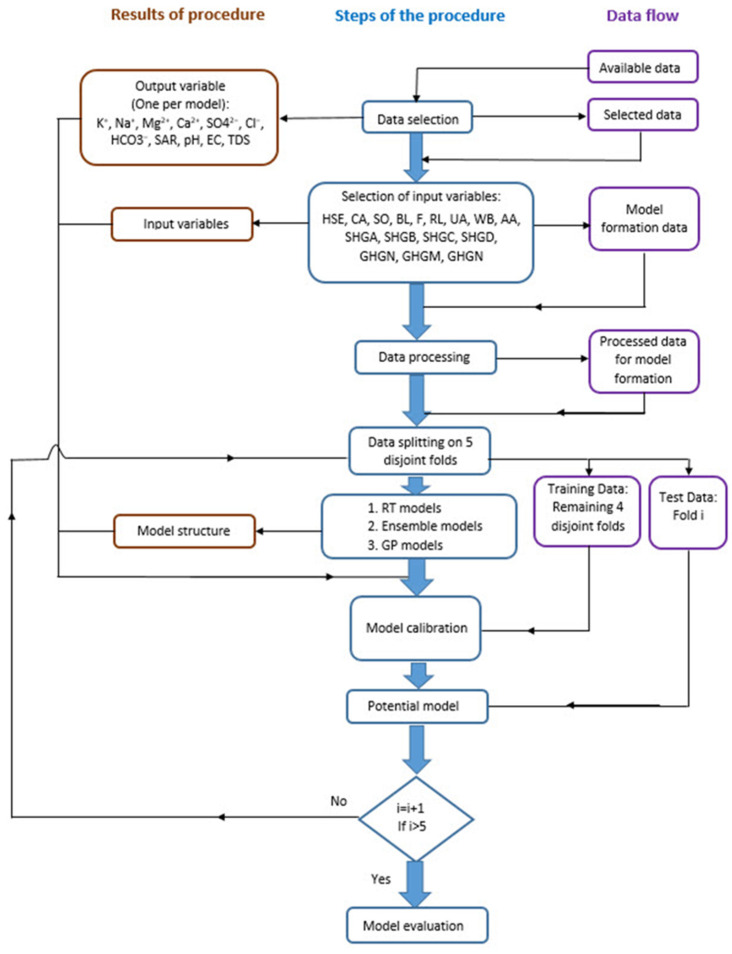
Applied methodology for creating prediction models.

Moreover, cross-validation provides a more robust estimate of the model’s performance by averaging the evaluation metrics across multiple folds. This helps ensure that our results are not overly dependent on the particular random split of the data. Additionally, cross-validation allows us to iteratively train and evaluate the model on different subsets, aiding in the fine-tuning of hyperparameters and ensuring the model’s performance is consistently reliable (Figure 5).

## 4. Results

The paper analyzes the application of regression trees, bagging, RF, gradient boosting, and Gaussian process regression models using a systemic approach (Figure 5). For each of the models, the hyperparameters of the model were varied in the appropriate range, and optimal values were determined using a grid-search method. The following values were analyzed:

- Regression trees (RT) model

The depth of a regression tree is a crucial factor in preventing overfitting, which occurs when the tree becomes too detailed and fits the training data too closely, as well as underfitting, which happens when the tree is too simplistic and fails to capture the underlying patterns. Table 3 illustrates the impact of the minimum leaf size on the accuracy of the regression tree (RT) model. It shows how changing the minimum leaf size affects key performance metrics such as RMSE, MAE, MAPE, and R. Accordingly, the leaf size is almost positively associated with the RMSE but inversely correlated with the R values.

- TreeBagger (TB) model
  1. Number of generated trees (B): Investigated values up to a maximum of 500 trees, with 100 as the standard setting in Matlab. The bootstrap aggregation method was employed, generating a specific number of samples in each iteration. The study considered an upper limit of 500 trees.
  2. Minimum amount of data/samples per tree leaf: Analyzed values ranging from 2 to 15 samples per leaf, with a step size of 1 sample. The standard setting in Matlab is 5 samples per leaf for regression, but here, a broader range was examined to assess its impact on model generalization.

- Random Forest (RF) model:
  1. Number of generated trees (B): Analyzed within a range of 100–500 trees, with 100 as the standard setting in Matlab. Cumulative MSE values for all base models in the ensemble were presented. Bootstrap aggregation was used to create trees, generating 181 samples per iteration. The study explored an extended ensemble of up to 500 regression trees, aligning with recommended Random Forest practices.
  2. Number of variables used for tree splitting: Based on guidance, the study selected a subset of approximately $\sqrt{p}$ predictors for branching, where p is the number of input variables. With 16 predictors, this translated to a subset of 4 variables, but in those research, a wider number of variables, ranging from 1 to 16, is investigated.
  3. Minimum number of samples per leaf: the study considered values from 2 to 10 samples per tree leaf, with a 1-sample increment.

- Boosted tree model:
  1. Number of generated trees (B): analyzed within a range of 1–100 trees.
  2. Learning rate (λ): explored a range, including 0.001, 0.01, 0.1, 0.5, 0.75, and 1.0.
  3. Tree splitting levels (d): analyzed from 1 (a decision stump) to 2^7^ = 128 in an exponential manner.

- Gaussian process regression (GPR) model
  With the GPR method, the application of different kernel functions were explored:
  1. Exponential, quadratic exponential, Mattern 3/2, Mattern 5/2, rational quadratic.
  2. ARD Exponential, ARD quadratic exponential, ARD Mattern 3/2, ARD Mattern 5/2, ARD rational quadratic.

All models were evaluated in terms of optimality in terms of the mean square error, and then the optimal model obtained from all the analyzed ones was evaluated on the test data using the RMSE, MAE, MAPE, and R criteria.

In the paper, a detailed procedure is illustrated for determining the optimal model for the prediction of the SAR parameter. In contrast, for all other models for the prediction, it is given in a more concise form. Accompanying results for other models, except the SAR parameter, can be found in the Appendix A (Table A1, Table A2, Table A3, Table A4, Table A5, Table A6, Table A7, Table A8, Table A9 and Table A10) of the paper.

### 4.1. Prediction of SAR Parameter Values

- Regression tree models

Table 3 illustrates the impact of the minimum leaf size on the accuracy of the regression tree (RT) model. It shows how changing the minimum leaf size affects key performance metrics such as RMSE, MAE, MAPE, and R.

**Figure 6:**
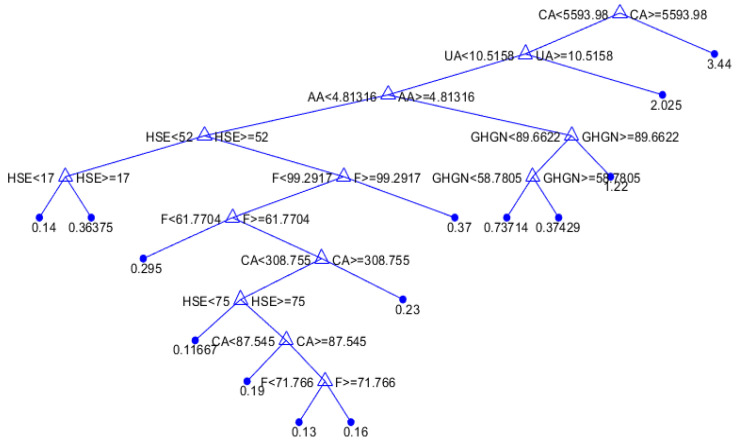
An optimal individual model for SAR parameter prediction based on a regression tree.

In this particular case, it was found that models with less complexity, i.e., the amount of data per terminal sheet is 10, have higher accuracy (Figure 6).

- TreeBagger models and Random Forest models

**Figure 7:**
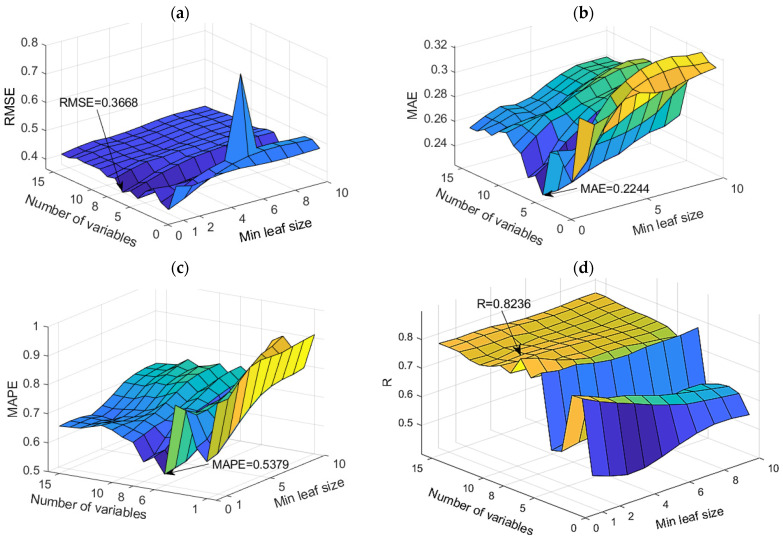
Comparison of different accuracy criteria for the RF model for the SAR parameter as a function of the number of randomly selected splitting variables and minimum leaf size: (**a**) RMSE, (**b**) MAE, (**c**) MAPE, (**d**) R.

The application of TB and RF models was analyzed simultaneously (Figure 7). The figure shows the dependence of the achieved accuracy of the model on the hyperparameter value. The TB model represents the borderline case of the RF model when all variables are taken into account for potential calculations.

Among the optimal models in this group, the RF model with 500 generated trees proved to be the best. In contrast, the model that uses a subset of eight variables and has a minimum amount of data per terminal leaf equal to one has a higher accuracy according to the RMSE and R criteria, while the model that uses a subset with six variables and has a minimum amount of data per terminal sheet equal to one and has a higher accuracy according to MAE and MAPE criteria (Table 4). Optimal values according to different accuracy criteria are marked with bold numbers in Table 4.

**Figure 8:**
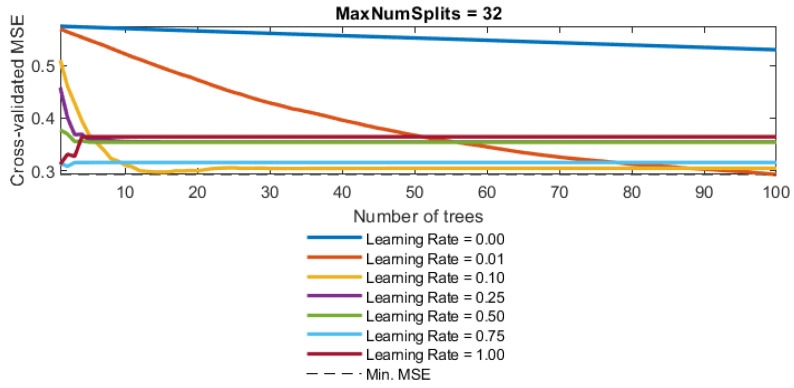
Dependence of the MSE value on the reduction parameter λ and the number of trees (base models) in the boosted tree model for the SAR parameter.

With the BT model (Figure 8), it was shown that the highest accuracy is obtained by applying complex models with a large number of branches.

The optimal obtained model had a structure of 32 branches and a Learning Rate value equal to 0.01.

- **GPR models**

The optimal values for the parameters of the applied models with different kernel functions were obtained using marine probability (Table 5 and Table 6).

The marginal likelihood is a function influenced by the observed data (y(X)) and model parameters {*l*, $\sigma^{2}$, $\eta^{2}$}. The determination of the model parameters is achieved through the maximization of this function.

Importantly, when the marginal likelihood is transformed by taking the logarithm, identical results are achieved as when optimizing the original likelihood. Therefore, model parameter optimization is typically carried out by employing gradient-based procedures on the log marginal probability expression, simplifying the optimization process without altering the final outcomes. The comparative results of the implemented ML models are presented in Table 7. Optimal values according to different accuracy criteria are marked with bold numbers in Table 7.

**Figure 9:**
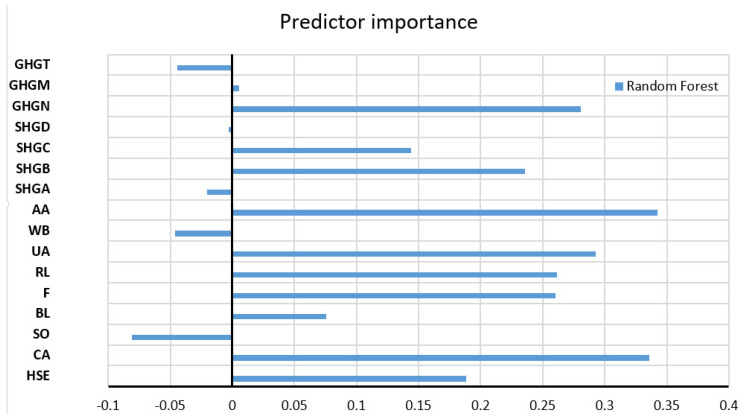
Significance of individual variables for SAR parameter prediction in an optimal RF model.

The values of all accuracy criteria according to the adopted accuracy criteria on the test data set are shown in Table 7. According to the RMSE and R criteria, the RF model had the highest accuracy (it uses a subset of eight variables for calculation, and the amount of data per terminal sheet is equal to one), while according to the MAE and MAPE criteria, the GP model with an exponential kernel function stood out as the most accurate. On the optimal RF model, the significance of each of the input variables was determined such that the values of the considered variable are permuted within the training data, and the out-of-bag error for such permuted data is recalculated. The significance of the variable (Figure 9) is then determined by calculating the mean value of the difference before and after a permutation. This value is then divided by the standard deviation of these differences. The variable for which a higher value was obtained in relation to the others is ranked as more significant in relation to the variables for which smaller values were obtained.

### 4.2. Prediction of Na^+^ Parameter Values

**Figure 10:**
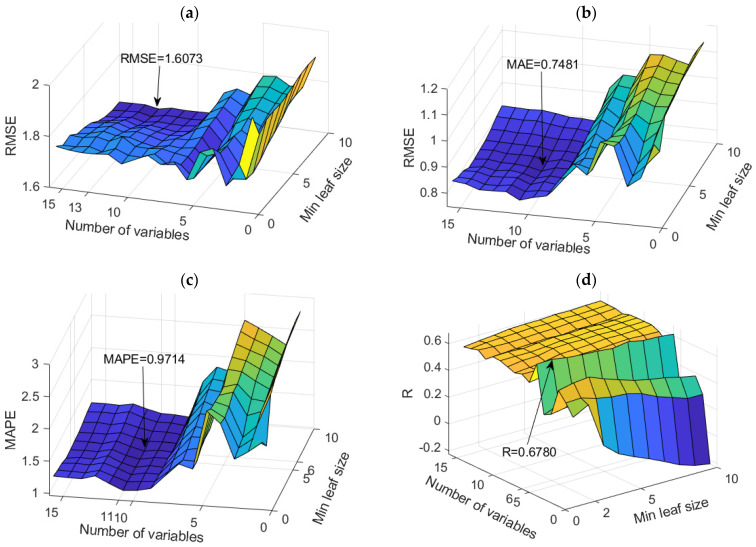
Comparison of different accuracy criteria for the RF model for the Na^+^ parameter as a function of the number of randomly selected splitting variables and minimum leaf size: (**a**) RMSE, (**b**) MAE, (**c**) MAPE, (**d**) R.

RF models proved to be the optimal models for predicting sodium ion (Na^+^) concentrations, while the analysis of all models in terms of accuracy is given in Appendix A (Table A1). The dependence of the adopted accuracy criteria on the model parameters is shown in Figure 10. Based on the defined accuracy criteria, four models with the following criteria values were selected (Table 8).

The “Weighted Sum Model” or “Simple Multi-Criteria Ranking” method was used to select the optimal model. For the minimization objectives (RMSE, MAE, MAPE), Min-Max normalization is applied, and for the maximization objective (R), Max-Min normalization is applied to ensure that all metrics are on the same scale. Equal weights are assigned to the normalized evaluation metrics to indicate their relative importance in the decision-making process. The weighted sum method calculated an aggregated value for each model, which considers all four normalized metrics. All models are ranked based on their aggregated values, with the lower aggregated value indicating better overall performance (Table 9).

**Figure 11:**
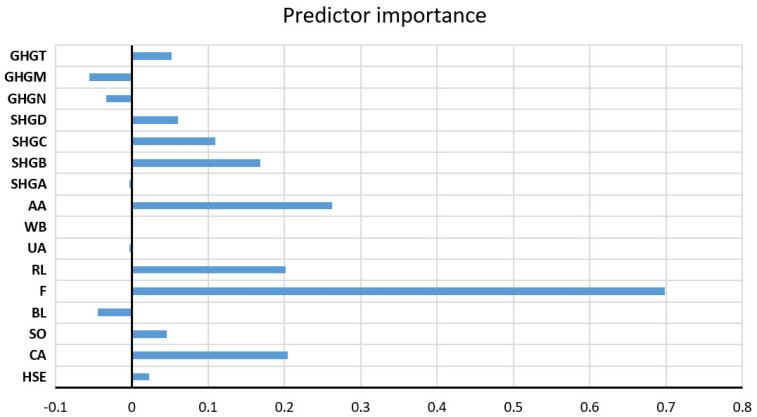
The significance of individual variables for Na^+^ parameter prediction in an optimal RF model.

As the optimal model, the RF model with 500 trees was obtained, which uses a subset of 11 variables, where the minimum amount of data per sheet is six. The assessment of the significance of individual input variables for the accuracy of the prediction was performed precisely on the obtained model with the highest accuracy (Figure 11).

### 4.3. Prediction of Magnesium (Mg^2+^) Parameter Values

**Figure 12:**
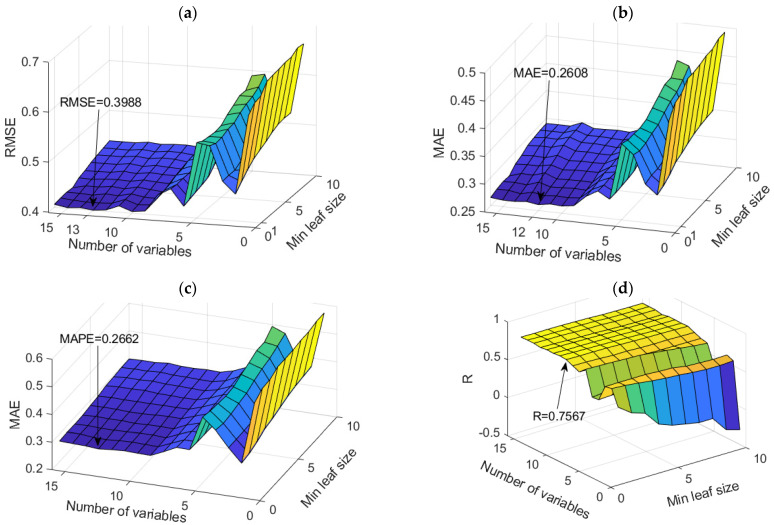
Comparison of different accuracy criteria for the RF model for the Mg^2+^ parameter as a function of the number of randomly selected splitting variables and minimum leaf size: (**a**) RMSE, (**b**) MAE, (**c**) MAPE, (**d**) R.

RF models proved to be the optimal models for predicting sodium ion (Mg^2+^) concentrations. An analysis of all models in terms of accuracy is given in Appendix A (Table A2). The dependence of the adopted accuracy criteria on the model parameters is shown in Figure 12. Based on the defined accuracy criteria, three models with the following values were selected (Table 10). Optimal values according to different accuracy criteria are marked with bold numbers in Table 10.

“Simple Multi-Criteria Ranking” was applied again when extracting the optimal model (Table 11).

**Figure 13:**
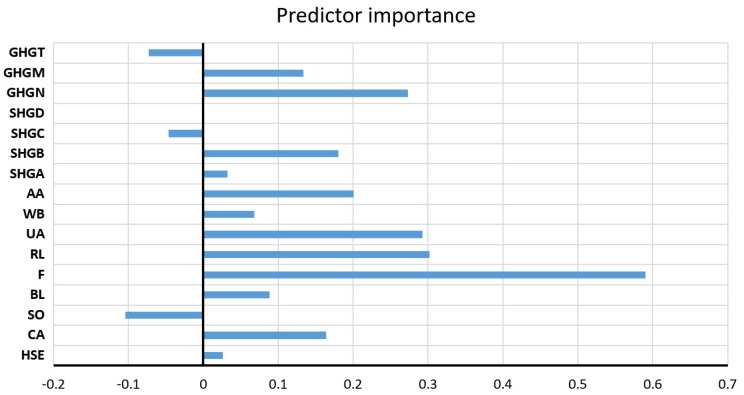
Significance of individual variables for Mg^2+^ parameter prediction in an optimal RF model.

As the optimal model, the RF model with 500 trees was obtained, which uses a subset of 12 variables, where the minimum amount of data per sheet is one. The assessment of the importance of individual input variables on the accuracy of the prediction was performed precisely on the obtained model with the highest accuracy (Figure 13).

### 4.4. Prediction of Ca^2+^ Parameter Values

**Figure 14:**
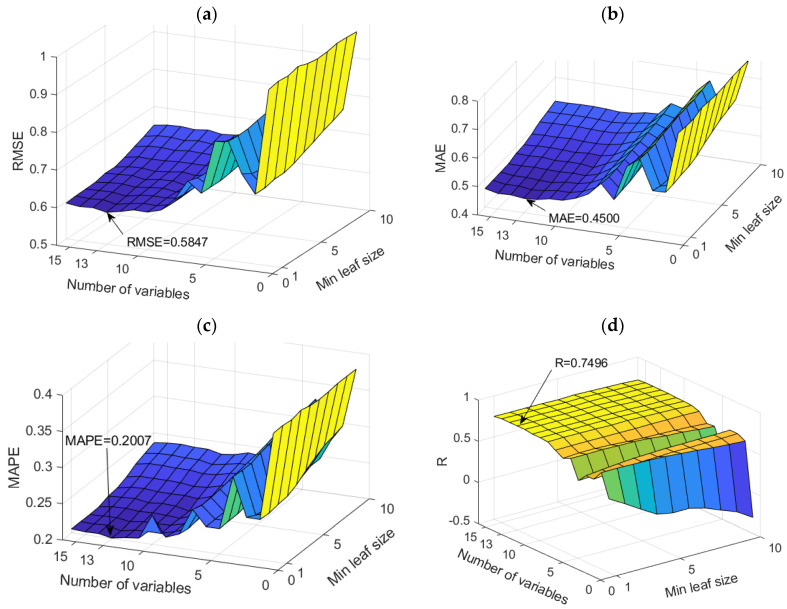
Comparison of different accuracy criteria for the RF model for the Ca^2+^ parameter as a function of the number of randomly selected splitting variables and minimum leaf size: (**a**) RMSE, (**b**) MAE, (**c**) MAPE, (**d**) R.

RF models proved to be the optimal models for Ca^2+^ (calcium ion concentration). An analysis of all models in terms of accuracy is given in Appendix A (Table A3). The dependence of the adopted accuracy criteria on the model parameters is shown in Figure 14. According to all the defined accuracy criteria, only one model stood out with values for RMSE, MAE, MAPE, and R of 0.5847, 0.4500, 0.2007, and 0.7496, respectively.

**Figure 15:**
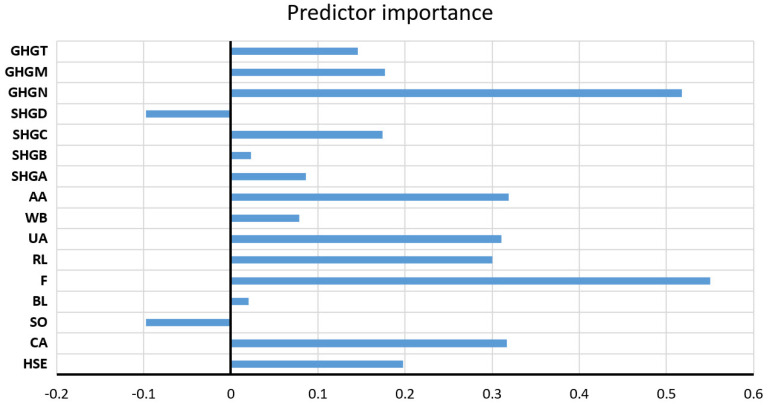
Significance of individual variables for Ca^2+^ parameter prediction in an optimal RF model.

The assessment of the significance of individual input variables on the accuracy of the prediction was performed precisely on the obtained model with the highest accuracy (Figure 15).

### 4.5. Prediction of SO_4_^2−^ Parameter Values

**Figure 16:**
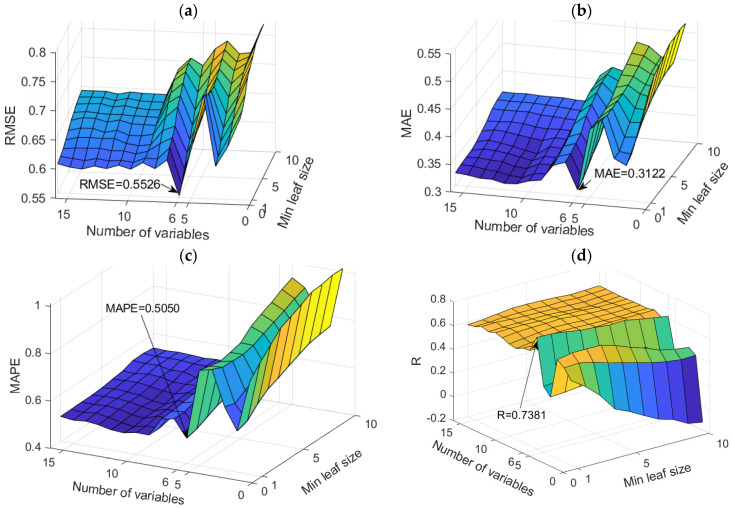
Comparison of different accuracy criteria for the RF model for the SO_4_^2−^ parameter as a function of the number of randomly selected splitting variables and minimum leaf size: (**a**) RMSE, (**b**) MAE, (**c**) MAPE, (**d**) R.

The RF models proved to be the optimal models for predicting SO_4_^2−^ levels. An analysis of all models in terms of accuracy is given in Appendix A (Table A4). The dependence of the adopted accuracy criteria on the model parameters is shown in Figure 16.

According to all defined accuracy criteria, only one model was singled out with values for RMSE, MAE, MAPE, and R of 0.5526, 0.3122, 0.5050, and 0.7381, respectively.

**Figure 17:**
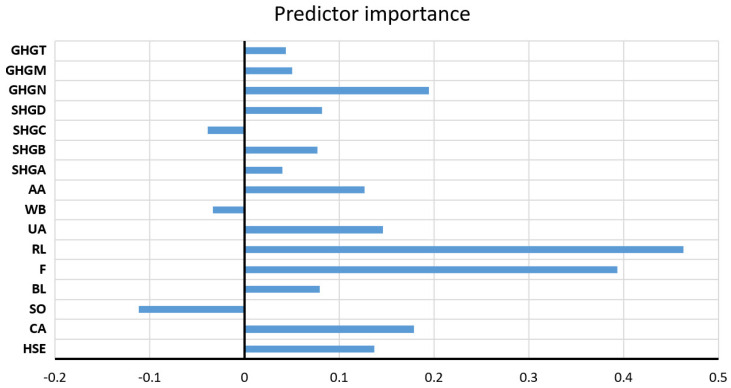
Significance of the individual variables for SO_4_^2−^ parameter prediction in an optimal RF model.

The assessment of the significance of the individual input variables for the accuracy of the prediction was performed directly on the obtained model with the highest accuracy (Figure 17).

### 4.6. Prediction of Cl^−^ Parameter Values

**Figure 18:**
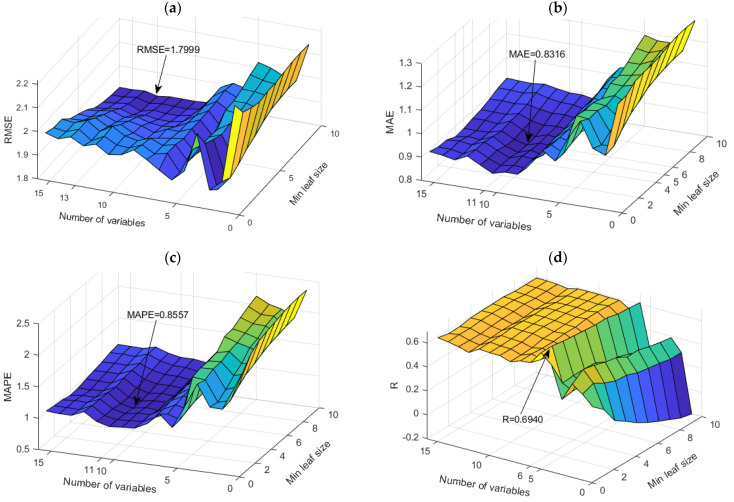
Comparison of different accuracy criteria for the RF model for the Cl^−^ parameter as a function of the number of randomly selected splitting variables and minimum leaf size: (**a**) RMSE, (**b**) MAE, (**c**) MAPE, (**d**) R.

RF models proved to be the optimal models for predicting Cl^−^ concentrations. An analysis of all models in terms of accuracy is given in Appendix A (Table A5). The dependence of the adopted accuracy criteria on the model parameters is shown in Figure 18. Based on the defined accuracy criteria, three models were selected (Table 12). Optimal values according to different accuracy criteria are marked with bold numbers in Table 12.

As the optimal model, the RF model with 500 trees was obtained, which uses a subset of 11 variables, where the minimum amount of data per leaf is five (Table 13).

**Figure 19:**
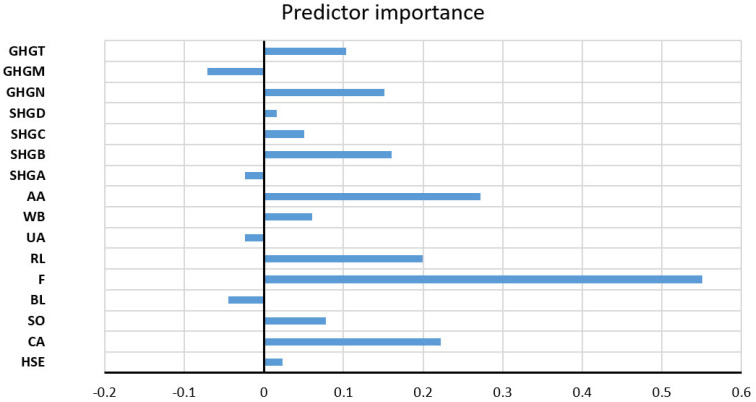
Significance of the individual variables for Cl^−^ parameter prediction in an optimal RF model.

The assessment of the importance of individual input variables on the accuracy of the prediction was performed precisely on the obtained model with the highest accuracy (Figure 19).

### 4.7. Prediction of HCO^3−^ Parameter Values

GPR models proved to be the optimal models for predicting HCO3− concentrations. Very similar values in terms of accuracy were also given by the RF models. However, since the difference between the GPR model and the RF model is practically negligible, and since it is not possible to obtain the significance value for individual input variables on the obtained GPR model because it has the same length scale parameter for all variables, RF models were used for the analysis. An analysis of all models in terms of accuracy is given in Appendix A (Table A6).

**Figure 20:**
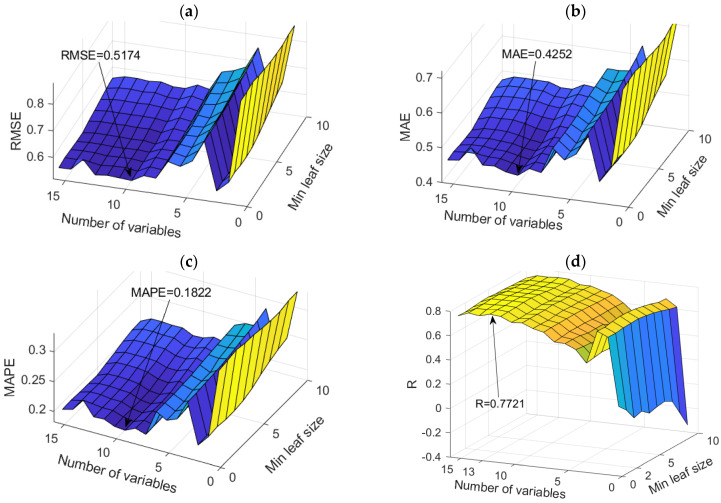
Comparison of different accuracy criteria for the RF model for the HCO^3−^ parameter as a function of the number of randomly selected splitting variables and minimum leaf size: (**a**) RMSE, (**b**) MAE, (**c**) MAPE, (**d**) R.

The dependence of the adopted accuracy criteria on the parameters of the RF model is shown in Figure 20.

In the specific case of applying the RF model, two models were distinguished, namely the RF model that uses ten variables as a subset for analysis and where the amount of data per terminal sheet is equal to one, which is optimal according to the RMSE, MAE, and MAPE criteria and the model that uses 13 variables as a subset for analysis and where the amount of data per terminal sheet is equal to two, which is optimal according to the R criterion. Since the first-mentioned model is optimal according to the three adopted accuracy criteria, RMSE, MAE, and MAPE, and the difference compared to the R criterion is practically negligible, the first model can be considered optimal.

The optimal model has the following criterion values for RMSE, MAE, MAPE, and R of 0.5174, 0.4252, 0.1822, and 0.7721, respectively.

**Figure 21:**
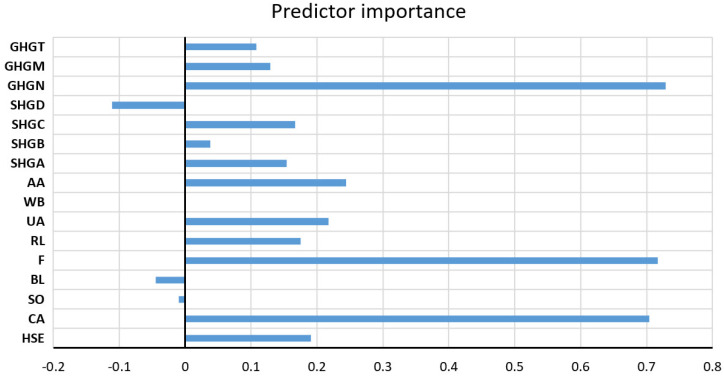
Significance of individual variables for HCO^3−^ parameter prediction in an optimal RF model.

The assessment of the importance of the individual input variables on the accuracy of the prediction was performed precisely on the obtained model with the highest accuracy (Figure 21).

### 4.8. Prediction of K^+^ Parameter Values

**Figure 22:**
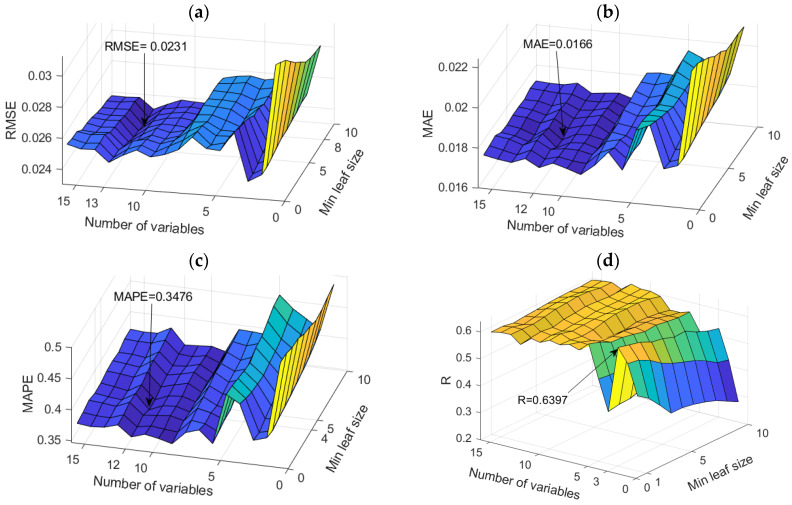
Comparison of different accuracy criteria for the RF model for the K+ parameter as a function of the number of randomly selected splitting variables and minimum leaf size: (**a**) RMSE, (**b**) MAE, (**c**) MAPE, (**d**) R.

The RF models proved to be the optimal models for predicting K^+^ levels. An analysis of all models in terms of accuracy is given in Appendix A (Table A7). The dependence of the adopted accuracy criteria on the model parameters is shown in Figure 22.

In terms of accuracy, three models were singled out, and the optimal model was obtained by applying the Simple Multi-Criteria Ranking method (Table 14 and Table 15). Optimal values according to different accuracy criteria are marked with bold numbers in Table 14.

**Figure 23:**
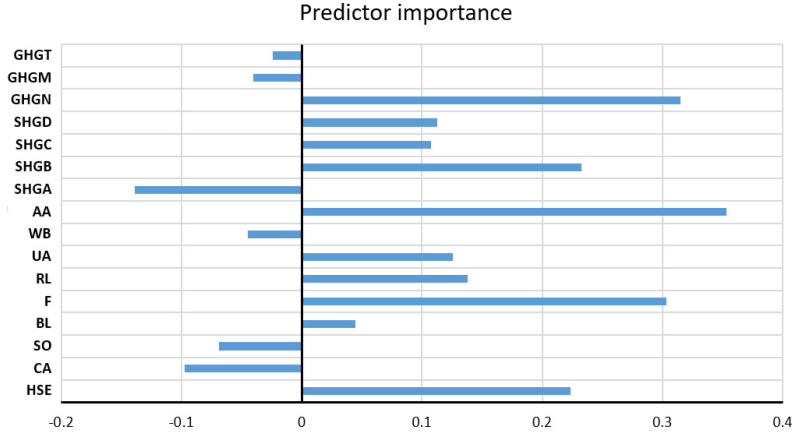
Significance of individual variables for K^+^ parameter prediction in an optimal RF model.

The analysis of the significance of the individual input variables of the model was performed on the optimal RF model with hyperparameter values for the value of the number of trees, a subset of variables for splitting, and the amount of data per terminal leaf, which are 500, 12, and 4, respectively (Table 15). The assessment of the importance of the individual input variables was performed precisely on the obtained model with the highest accuracy (Figure 23).

### 4.9. Prediction of pH Parameter Values

**Figure 24:**
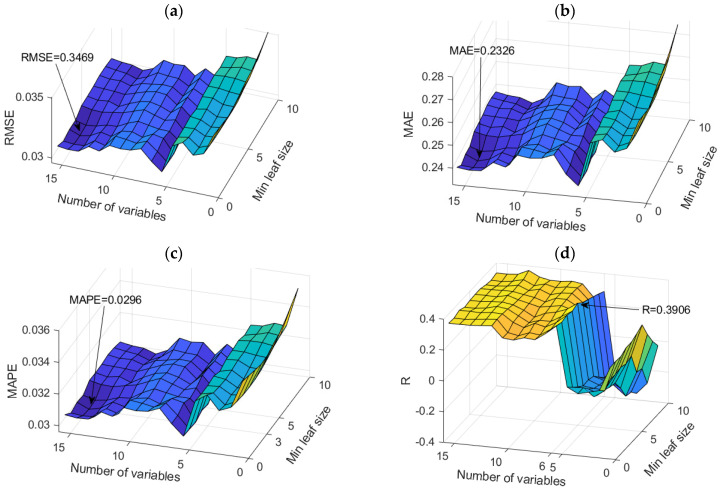
Comparison of different accuracy criteria for the RF model for the pH parameter as a function of the number of randomly selected splitting variables and minimum leaf size: (**a**) RMSE, (**b**) MAE, (**c**) MAPE, (**d**) R.

The RF models proved to be the optimal models for predicting SO_4_ levels. An analysis of all models in terms of accuracy is given in Appendix A (Table A8). The dependence of the adopted accuracy criteria on the model parameters is shown in Figure 24.

In terms of accuracy, three models were singled out, and the optimal model was obtained by applying the Simple Multi-Criteria Ranking method (Table 16 and Table 17). Optimal values according to different accuracy criteria are marked with bold numbers in Table 16.

Using the weighted sum method, an aggregated value for each model is calculated, which takes into account all four normalized metrics.

**Figure 25:**
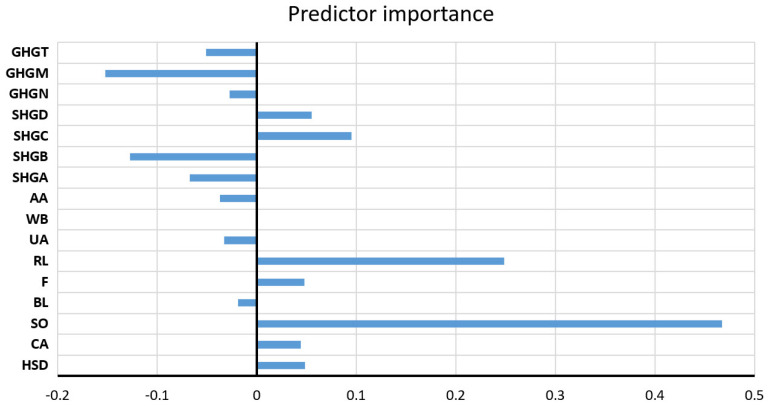
Significance of the individual variables for PH parameter prediction in an optimal RF model.

The analysis of the significance of the individual input variables of the model was performed on the optimal RF model and shown in Figure 25.

### 4.10. Prediction of EC Parameter Values

**Figure 26:**
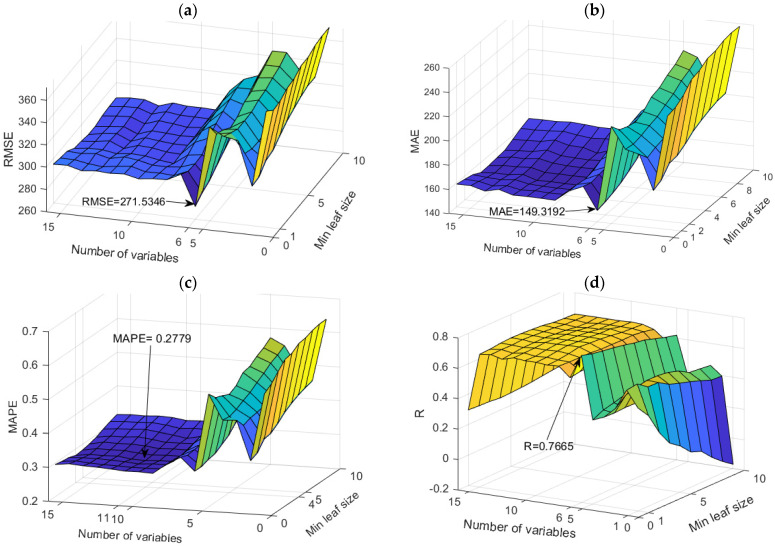
Comparison of different accuracy criteria for the RF model for the EC parameter as a function of the number of randomly selected splitting variables and minimum leaf size: (**a**) RMSE, (**b**) MAE, (**c**) MAPE, (**d**) R.

RF models proved to be optimal models for EC parameter prediction. An analysis of all models in terms of accuracy is given in Appendix A (Table A9). The dependence of the adopted accuracy criteria on the model parameters is shown in Figure 26.

According to all defined accuracy criteria, only one model was singled out with values for RMSE, MAE, MAPE, and R of 271.5346, 149.3192, 0.2779, and 0.7665, respectively.

**Figure 27:**
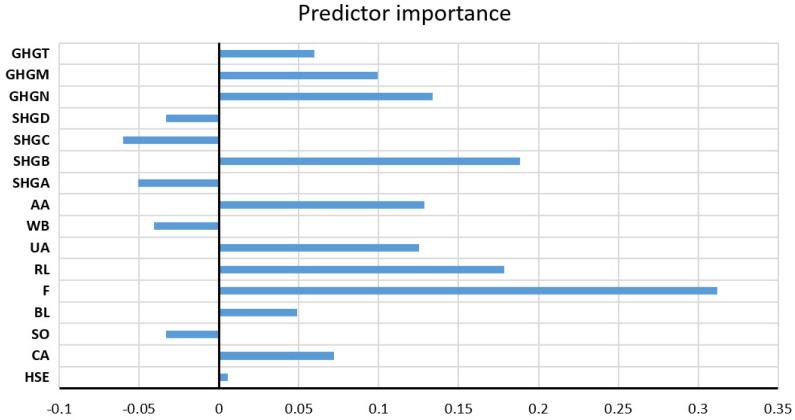
Significance of the individual variables for EC parameter prediction in an optimal RF model.

The obtained hyperparameter values for the number of trees, the subset of splitting variables, and the minimum amount of data per leaf are 500, 6, and 1, respectively. The analysis of the significance of the individual input variables of the model was performed on the optimal RF model and shown in Figure 27.

### 4.11. Prediction of TDS Parameter Values

**Figure 28:**
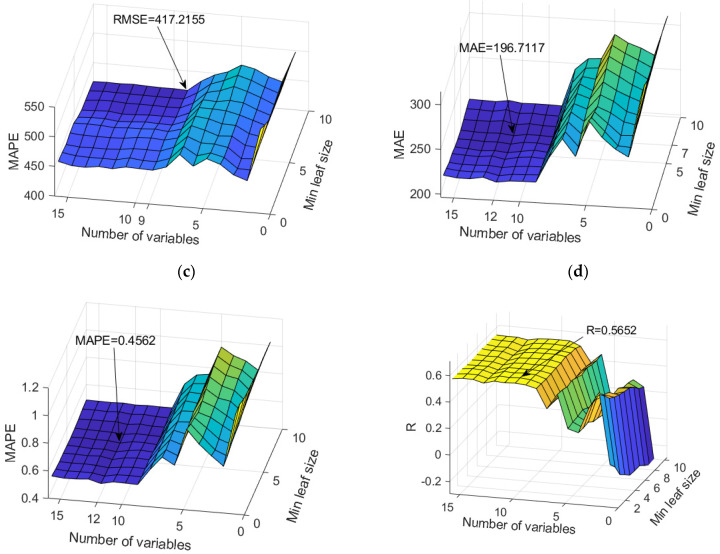
Comparison of different accuracy criteria for the RF model for the TDS parameter as a function of the number of randomly selected splitting variables and minimum leaf size: (**a**) RMSE, (**b**) MAE, (**c**) MAPE, (**d**) R.

The RF models proved to be the optimal models for predicting SO_4_^2−^ levels. An analysis of all models in terms of accuracy is given in Appendix A (Table A10). The dependence of the adopted accuracy criteria on the model parameters is shown in Figure 28.

In terms of accuracy, three models were singled out, and the optimal model was obtained by applying the Simple Multi-Criteria Ranking method (Table 18 and Table 19). Optimal values according to different accuracy criteria are marked with bold numbers in Table 18.

**Figure 29:**
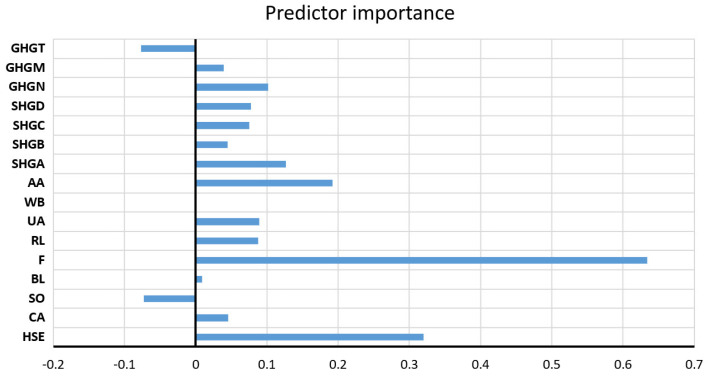
Significance of the individual variables for TDS parameter prediction in an optimal RF model.

The analysis of the significance of the individual input variables of the model was performed on the optimal RF model and shown in Figure 29.

## 5. Discussion

In our research, most models demonstrated satisfactory accuracy, meeting the predefined criteria. However, a subset of models exhibited shortcomings in specific criteria. To gauge accuracy effectively, we leaned on relative metrics, notably accuracy (R) and mean absolute percentage error (MAPE), as they offer more insightful perspectives compared to absolute criteria such as RMSE and MAE (Table 20).

Table 20 highlights the accuracy of the machine learning models in predicting individual water parameters. Notably, the RF model emerged as the best performer across various parameters, underscoring its efficacy.

Analyzing the R values reveals the overall satisfactory performance of most models, except for the pH prediction model. Examining MAPE values identified five models—SAR, Na+, SO_4_, Cl, and TDS—where this metric is relatively higher than other ones. Despite these nuances, our primary research focus was unraveling the significance of individual input variables within the constraints of limited data.

When we delve into the significance of the individual input variables, our conclusions (Table 21) unveil the following crucial insights:

Forest Cover (‘F’): Forest areas significantly influence diverse water quality parameters. Trees and vegetation in forests contribute organic matter to water bodies, influencing ion concentrations. The root systems of trees can affect the uptake of certain ions. Forests strongly impact the concentrations of sodium, magnesium, calcium, chloride, sulfate, bicarbonate, and potassium ions. Also, forests act as natural filters, reducing the transport of sediments and pollutants into water bodies. Cleaner water, with fewer suspended solids, tends to have lower TDS and EC. Additionally, forest areas often have minimal human activities compared to urban or agricultural areas.

Rangeland (RL) is essential for predicting water sulfate ion concentrations. This suggests that the characteristics associated with the rangeland, such as land cover and land use patterns, significantly influence sulfate levels. Additionally, rangeland strongly affects SAR by influencing sodium concentrations, vital for evaluating water’s suitability for irrigation and soil health. Also, the notable impact on magnesium levels showcases rangeland’s role in shaping water quality. Rangeland’s influence on pH highlights its role in determining water acidity or alkalinity, which is crucial for aquatic ecosystems and nutrient availability. Additionally, rangeland significantly influences electrical conductivity, providing insights into water quality and dissolved ion content, essential for understanding overall water composition. While having a somewhat lesser impact, rangeland still plays a discernible role in shaping sodium concentrations, contributing to insights into water salinity and its ecological implications.

Urban Area (‘UA’): Urban areas have a moderate impact on ion levels, magnesium, chloride, bicarbonate, and SAR parameters, owing to urbanization and land use changes, introducing contaminants and altering water chemistry. Calcium, sulfate, and EC parameters have less impact.

The Agricultural Area (AA) substantially impacts potassium, SAR, and sodium, with a moderate impact on calcium, TDS, and magnesium. The influence of AA on these parameters can be explained by the agricultural areas’ use of potassium-containing fertilizers, leading to elevated potassium concentrations in water. Cultivation practices and nutrient management contribute to increased potassium levels. Additionally, agricultural activities often involve irrigation, and water with high sodium content can increase SAR. Sodium in the soil can be introduced through irrigation water, affecting sodium levels in the water. Moreover, agricultural runoff can introduce calcium, magnesium, and other dissolved solids into water sources.

Catchment Area (‘CA’): The size of catchment areas plays a moderate role in ion transport, particularly affecting SAR, sodium, bicarbonate, calcium, and sulfate levels. The size of the catchment area could moderately impact SAR, as larger areas may interact with more diverse geological and soil features, affecting sodium adsorption ratios.

Considering different soil types (HSGA, HSGB, HSGC, HSGD) and geological permeability (GHGM, GHGN, GHGT) underscores their impact on ion retention and release. Sandy soils facilitate easier ion movement, while clayey soils retain ions. Geological permeability influences potassium, magnesium, calcium, and bicarbonate levels, showcasing the interconnectedness of soil and geological characteristics with water parameters.

## 6. Conclusions

Our study demonstrates the effectiveness of machine learning methods in predicting and assessing water quality parameters within a catchment area. With the Random Forest (RF) model as the standout performer, the model provides a robust tool for efficient and accurate water quality evaluation.

While certain models may fall short on specific criteria, a nuanced evaluation leveraging relative criteria like accuracy (R) and mean absolute percentage error (MAPE) underscores the overall robustness of the predictive models. Table 20 encapsulates the detailed results, highlighting the efficacy of the RF model across various water parameters.

Evaluation of R values showcases all models’ satisfactory performance except for pH prediction. Despite marginally elevated MAPE values in five models (SAR, Na+, SO_4_, Cl, TDS), the core research objective—unraveling the importance of individual input variables within data constraints—was largely achieved.

This accomplishment paves the way for selecting and implementing optimal models from a broader ML spectrum. To further elevate model accuracy, future research will focus on dataset expansion, a strategic initiative to address current limitations and achieve heightened accuracy, particularly in parameters exhibiting slight deviations.

The significance of individual input variables, as outlined in Table 21, provides crucial insights for understanding their roles in influencing water parameters. Forest cover, catchment area characteristics, stream order, barren land, and urban areas are pivotal factors shaping water quality.

Incorporating these research insights into decision-making processes presents transformative opportunities for strategic resource allocation and environmental impact mitigation. Furthermore, integrating these outcomes empowers decision-makers to adopt targeted strategies for fostering environmental sustainability, contributing to the broader goal of cultivating resilient water ecosystems. This integration signifies a practical pathway toward achieving a delicate balance between human activities and environmental preservation, actively contributing to sustainable water ecosystems.

## Figures and Tables

**Table 1:** Statistical properties of the input variables used for modeling.

Input Parameter (Acronym)	Min	Max	Average	Std
Hydrometric Station Elevation (HSE)	−23.0000	2360.0000	163.3333	363.3326
Catchment Area (CA)	22.0300	6328.2800	422.7320	1085.4846
Stream Order (SO)	1.0000	4.0000	2.6275	1.3261
Percentage of Land Use or Land Cover Types:				
Barren Land (BL)	0.0000	3.1825	0.1246	0.6083
Forest (F)	1.1805	100.0000	70.0401	29.5955
Rangeland (RL)	0.0000	90.3170	17.0144	23.9666
Urban Area (UA)	0.0000	20.2095	1.1367	3.5475
Water Body (WB)	0.0000	0.3567	0.0074	0.0499
Agricultural Area (AA)	0.0000	84.3857	11.6768	20.3896
Hydrological Soil Group:				
A—Sand, loamy sand, or sandy loam (HSGA)	0.0000	79.3654	8.0039	16.2217
B—Silt loam or loam (HSGB)	0.0000	48.4653	3.0354	8.4226
C—Sandy clay loam (HSGC)	12.9196	100.0000	80.4068	27.0174
D—Clay loam, silty clay loam, sandy clay, silty clay, or clay (HSGD)	0.0000	56.4129	8.5539	15.9743
Geological Permeability:				
Low (Geological hydrological group M—GHGM)	0.0143	100.0000	69.2656	28.0000
Average (Geological hydrological group N—GHGN)	0.0000	96.9436	23.4102	24.3102
High (Geological hydrological group T—GHGT)	0.0000	90.9015	7.3243	15.3979

**Table 2:** Statistical properties of the output variables used for modeling.

Parameter	Min	Max	Average	Std
SAR	7.1500	9.0900	7.5318	0.3976
Na^+^	0.1200	15.8400	0.9978	2.3957
Mg^2+^	0.4100	4.4100	1.0331	0.6790
Ca^2+^	1.0600	5.8800	2.3584	0.9521
SO_4_^2−^	0.2100	4.4500	0.6643	0.8449
Cl^−^	0.1900	18.2000	1.1861	2.7131
HCO^3−^	1.3500	4.0900	2.5978	0.7729
pH	172.0500	2879.9700	453.7716	428.3365
EC	108.8900	3892.8200	375.2543	579.1442
TDS	0.1000	3.4400	0.4750	0.5971
K^+^	0.0200	0.1400	0.0447	0.0316

**Table 3:** Influence of the minimum leaf size on regression tree (RT) model accuracy.

Min Leaf Size	RMSE	MAE	MAPE	R
1	0.5646	0.2753	0.5948	0.4363
2	0.6077	0.2923	0.6322	0.3226
3	0.6096	0.2875	0.6311	0.2909
4	0.5984	0.2914	0.6188	0.3186
5	0.5894	0.2931	0.6728	0.3621
6	0.5833	0.2925	0.6616	0.3593
7	0.5813	0.2949	0.6968	0.3542
8	0.5841	0.3095	0.7603	0.3226
9	0.5821	0.2990	0.7078	0.3432
10	0.5969	0.3083	0.7369	0.2906

**Table 4:** Accuracy of obtained models for SAR parameter prediction according to defined criteria.

Criteria	RMSE	MAE	MAPE	R
RF 1 (var 8, leaf 1)	**0.3668**	0.2328	0.5679	**0.8236**
RF 2 (var 6, leaf 1)	0.3696	**0.2244**	0.5379	0.8012

**Table 5:** Values of optimal parameters in GPR models with different covariance functions.

GP Model Covariance Function	Covariance Function Parameters			
Exponential	$k\left(\left(x_{i},x_{j}|\Theta \right)\right)=\sigma_{f}^{2}exp\left[-\frac{1}{2}\frac{r}{{\sigma_{l}}^{2}}\right]$			
	$\sigma_{l}=$ 111.9371		$\sigma_{f}=$ 1.8001	
Squared Exponential	$k\left(\left(x_{i},x_{j}|\Theta \right)\right)=\sigma_{f}^{2}exp\left[-\frac{1}{2}\frac{{\left(x_{i}-x_{j}\right)}^{T}\left(x_{i}-x_{j}\right)}{{\sigma_{l}}^{2}}\right]$			
	$\sigma_{l}=$ 8.3178		$\sigma_{f}=$ 1.2040	
Matern 3/2	$k\left(\left(x_{i},x_{j}|\Theta \right)\right)=\sigma_{f}^{2}\left(1+\frac{\sqrt{3}r}{\sigma_{l}}\right)exp\left[-\frac{\sqrt{3}r}{\sigma_{l}}\right]$			
	$\sigma_{l}=$14.7080		$\sigma_{f}=$1.4023	
Matern 5/2	$k\left(\left(x_{i},x_{j}|\Theta \right)\right)=\sigma_{f}^{2}\left(1+\frac{\sqrt{5}r}{\sigma_{l}}+\frac{5r^{2}}{3{\sigma_{l}}^{2}}\right)exp\left[-\frac{\sqrt{5}r}{\sigma_{l}}\right]$			
	$\sigma_{l}=$ 9.9890		$\sigma_{f}=$ 1.1947	
Rational Quadratic	$k\left(\left(x_{i},x_{j}|\Theta \right)\right)=\sigma_{f}^{2}{\left(1+\frac{r^{2}}{2a{\sigma_{l}}^{2}}\right)}^{-\alpha };r=0$			
	$\sigma_{l}=$ 8.3178	a= 3,156,603.8854		$\sigma_{f}=$ 1.2040

where $r=\sqrt{{\left(x_{i}-x_{j}\right)}^{T}\left(x_{i}-x_{j}\right)}$.

**Table 6:** Values of optimal parameters in GPR ARD models with different covariance functions.

Covariance Function Parameters															
$\sigma_{1}$	$\sigma_{2}$	$\sigma_{3}$	$\sigma_{4}$	$\sigma_{5}$	$\sigma_{6}$	$\sigma_{7}$	$\sigma_{8}$	$\sigma_{9}$	$\sigma_{10}$	$\sigma_{11}$	$\sigma_{12}$	$\sigma_{13}$	$\sigma_{14}$	$\sigma_{15}$	$\sigma_{16}$
ARD Exponential: $k\left(\left(x_{i},x_{j}|\Theta \right)\right)=\sigma_{f}^{2}\exp\left(-r\right)$; $\sigma_{F}=1$.; $r=\sqrt{\sum_{m=1}^{d}\frac{{\left(x_{im}-x_{jm}\right)}^{2}}{{\sigma_{m}}^{2}}}$															
119.8634	29.7051	300.3391	$7.6459\times 10^{5}$	158.5921	$1.0859\times 10^{7}$	15.3385	6.9058	89.5438	109.1181	121.9050	$3.5665\times 10^{5}$	$3.6154\times 10^{6}$	114.0696	$5.0314\times 10^{5}$	245.9937
ARD Squared exponential: $k\left(\left(x_{i},x_{j}|\Theta \right)\right)=\sigma_{f}^{2}exp\left[-\frac{1}{2}\sum_{m=1}^{d}\frac{{\left(x_{im}-x_{jm}\right)}^{2}}{{\sigma_{m}}^{2}}\right];$ $\sigma_{f}=1.0577$															
0.9131	4.9648	$3.2490\times 10^{11}$	166.9588	$3.6087\times 10^{6}$	$4.9117\times 10^{6}$	2.3307	1.1875	11.1584	14.6207	15.1518	$1.4141\times 10^{6}$	32.6135	$3.4365\times 10^{9}$	$1.5821\times 10^{5}$	$2.2696\times 10^{4}$
ARD Matern 3/2: $k\left(\left(x_{i},x_{j}|\Theta \right)\right)=\sigma_{f}^{2}\left(1+\sqrt{3}r\right)exp\left[-\sqrt{3}r\right];$ $\sigma_{f}=1.3295$															
$2.3961\times 10^{5}$	9.2294	$1.3527\times 10^{9}$	$9.4195\times 10^{16}$	32.1227	$9.4054\times 10^{8}$	9.6482	4.7958	$1.1130\times 10^{5}$	28.2837	4.3381	$7.8940\times 10^{4}$	131.4068	62.5967	$1.7599\times 10^{6}$	$7.2779\times 10^{10}$
ARD Matern 5/2: $k\left(\left(x_{i},x_{j}|\Theta \right)\right)=\sigma_{f}^{2}\left(1+\sqrt{5}r+\frac{5r^{2}}{3}\right)exp\left[-\sqrt{5}r\right];$ $\sigma_{f}=1.0452$															
0.2981	$3.4788\times 10^{4}$	27.1402	16.2408	$6.4079\times 10^{4}$	16.8653	3.3999	3.4057	$9.1495\times 10^{4}$	11.2644	$6.3690\times 10^{5}$	$1.4039\times 10^{6}$	30.9904	7.7608	23.1035	33.8984
ARD Rational quadratic: $k\left(\left(x_{i},x_{j}|\Theta \right)\right)=\sigma_{f}^{2}{\left(1+\frac{1}{2\alpha }\sum_{m=1}^{d}\frac{{\left(x_{im}-x_{jm}\right)}^{2}}{{\sigma_{m}}^{2}}\right)}^{-\alpha };\alpha$ = 0.7452; $\sigma_{f}=1.07$															
$2.4546\times 10^{6}$	3.7479	$2.3486\times 10^{7}$	$5.3012\times 10^{7}$	$3.9435\times 10^{3}$	10.1652	2.2446	0.7733	7.0275	18.4361	13.7803	$1.3226\times 10^{7}$	30.2548	$5.2024\times 10^{5}$	41.8960	$7.2334\times 10^{7}$

where $r=\sqrt{\sum_{m=1}^{d}\frac{{\left(x_{im}-x_{jm}\right)}^{2}}{{\sigma_{m}}^{2}}}$.

**Table 7:** Comparative analysis of the results of different machine learning models for the SAR prediction.

Model	RMSE	MAE	MAPE/100	R
Decision Tree	0.5646	0.2753	0.5948	0.4363
TreeBagger	0.4021	0.2513	0.6413	0.7652
RF 1 (var 8, leaf 1)	**0.3668**	0.2328	0.5679	**0.8236**
RF 2 (var 6, leaf 1)	0.3696	0.2244	0.5379	0.8012
Boosted Trees	0.5592	0.3348	0.6047	0.5867
GP exponential	0.4625	**0.2104**	**0.4998**	0.6317
GP Sq. exponential	0.4868	0.2393	0.5810	0.5733
GP matern 3/2	0.4757	0.2293	0.5406	0.5992
GP matern 5/2	0.4779	0.2307	0.5520	0.5941
GP Rat. quadratic	0.4868	0.2393	0.5810	0.5733
GP ARD exponential	0.5917	0.2873	0.6991	0.3302
GP ARD Sq. exponential	0.5669	0.2788	0.7736	0.3568
GP ARD matern 3/2	0.5276	0.2707	0.7206	0.4702
GP ARD matern 5/2	0.5464	0.2875	0.8794	0.4223
GP ARD Rat. quadratic	0.6573	0.3285	0.9059	0.2349

**Table 8:** Accuracy of obtained models for Na^+^ parameter prediction according to defined criteria.

Criteria	RMSE	MAE	MAPE	R
RF 1 (var 13, leaf 10)	1.6073	0.8086	1.1651	0.5817
RF 2 (var 11, leaf 5)	1.6755	0.7481	0.9734	0.5919
RF 3 (var 11, leaf 6)	1.6595	0.7516	0.9714	0.5923
RF 4 (var 6, leaf 2)	1.6385	0.8772	1.3929	0.6780

**Table 9:** Determining the optimal prediction model for the Na^+^ parameter using Simple Multi-Criteria Ranking.

Weighted Criteria	w.RMSE	w.MAE	w.MAPE	w.R	Agg. Value
RF 1 (var 13, leaf 10)	0.2500	0.1328	0.1351	0.0000	0.5180
RF 2 (var 11, leaf 5)	0.0000	0.2500	0.2488	0.0265	0.5253
**RF 3 (var 11, leaf 6)**	0.0587	0.2432	0.2500	0.0275	0.5794
RF 4 (var 6, leaf 2)	0.1356	0.0000	0.0000	0.2500	0.3856

**Table 10:** The accuracy of the obtained models for Mg^2+^ prediction according to defined criteria.

Criteria	RMSE	MAE	MAPE	R
RF 1 (var 13, leaf 1)	**0.3988**	0.2640	**0.2662**	0.7377
RF 2 (var 12, leaf 1)	0.4014	**0.2608**	0.2706	0.7516
RF 3 (var 10, leaf 1)	0.4020	0.2631	0.2717	**0.7567**

**Table 11:** Determining the optimal prediction model for the Mg^2+^ parameter using Simple Multi-Criteria Ranking.

Weighted Criteria	w.RMSE	w.MAE	w.MAPE	w.R	Agg. Value
RF 1 (var 13, leaf 1)	0.2500	0.0000	0.2500	0.0000	0.5000
**RF 2 (var 12, leaf 1)**	0.0469	0.2500	0.0500	0.1829	0.5298
RF 3 (var 10, leaf 1)	0.0000	0.0703	0.0000	0.2500	0.3203

**Table 12:** Accuracy of the obtained models for Cl^−^ prediction according to defined criteria.

RF Model	RMSE	MAE	MAPE	R
RF 1 (var 13, leaf 10)	**1.7999**	0.9111	1.1120	0.5691
RF 2 (var 11, leaf 5)	1.8831	**0.8316**	0.8589	0.5964
RF 3 (var 11, leaf 4)	1.8904	0.8323	**0.8557**	0.5933
RF 4 (var 6, leaf 2)	1.8473	0.9370	1.0288	**0.6940**

**Table 13:** Determining the optimal prediction model for the Cl^−^ parameter using Simple Multi-Criteria Ranking.

RF Model	w.RMSE	w.MAE	w.MAPE	w.R	Agg. Value
RF 1 (var 13, leaf 10)	0.2500	0.0614	0.0000	0.0000	0.3114
**RF 2 (var 11, leaf 5)**	0.0202	0.2500	0.2469	0.0546	0.5717
RF 3 (var 11, leaf 4)	0.0000	0.2483	0.2500	0.0484	0.5468
RF 4 (var 6, leaf 2)	0.1191	0.0000	0.0812	0.2500	0.4502

**Table 14:** Accuracy of obtained models for K^+^ parameter prediction according to defined criteria.

Criteria	RMSE	MAE	MAPE	R
Var 13, leaf 8	**0.0231**	0.0172	0.3755	0.5689
Var 3, leaf 1	0.0236	0.0174	0.3700	**0.6397**
Var 12, leaf 4	0.0241	**0.0166**	**0.3476**	0.6024

**Table 15:** Determining the optimal prediction model for the K^+^ parameter using Simple Multi-Criteria Ranking.

Weighted Criteria	w.RMSE	w.MAE	w.MAPE	w.R	Agg. Value
Var 13, leaf 8	0.2500	0.0625	0.0000	0.0000	0.3125
Var 3, leaf 1	0.1250	0.0000	0.0493	0.2500	0.4243
**Var 12, leaf 4**	0.0000	0.2500	0.2500	0.1183	0.6183

**Table 16:** Accuracy of the obtained models for pH parameter prediction according to defined criteria.

Criteria	RMSE	MAE	MAPE	R
RF 1 (var 7, leaf 1)	**0.3469**	0.2383	0.0306	0.3331
RF 2 (var 15, leaf 3)	0.3554	**0.2326**	**0.0296**	0.3476
RF 3 (var 6, leaf 5)	0.3531	0.2338	0.0298	**0.3906**

**Table 17:** Determining the optimal prediction model for the PH parameter using Simple Multi-Criteria Ranking.

Weighted Criteria	w.RMSE	w.MAE	w.MAPE	w.R	Agg. Value
RF 1 (var 7, leaf 1)	0.2500	0.0000	0.0000	0.0000	0.2500
RF 2 (var 15, leaf 3)	0.0000	0.2500	0.2500	0.0630	0.5630
**RF 3 (var 6, leaf 5)**	0.0676	0.1974	0.2000	0.2500	0.7150

**Table 18:** Accuracy of the obtained models for TDS parameter prediction according to defined criteria.

Criteria	RMSE	MAE	MAPE	R
RF 1 (Var 9, leaf 10)	**417.2155**	201.8572	0.4863	0.5467
RF 1 (Var 12, leaf 7)	422.6822	**196.7117**	0.4578	**0.5521**
RF 1 (Var 12, leaf 5)	435.3533	198.3639	**0.4562**	0.5502

**Table 19:** Determining the optimal prediction model for the TDS parameter using Simple Multi-Criteria Ranking.

Weighted Criteria	w.RMSE	w.MAE	w.MAPE	w.R	Agg. Value
RF 1 (Var 9, leaf 10)	0.2500	0.0000	0.0000	0.0000	0.2500
**RF 1 (Var 12, leaf 7)**	0.1747	0.2500	0.2367	0.2500	0.9114
RF 1 (Var 12, leaf 5)	0.0000	0.1697	0.2500	0.1620	0.5818

**Table 20:** Accuracy of the ML model in predicting individual water parameters.

Output Parameter	Best Model	RMSE	MAE	MAPE	R
SAR	RF	0.3668	0.2328	0.5679	0.8236
Na^+^	RF	16.385	0.8772	13.929	0.678
Mg^2+^	RF	0.402	0.2631	0.2717	0.7567
Ca^2+^	RF	0.5847	0.45	0.2007	0.7496
SO_4_^2−^	RF	0.5526	0.3122	0.505	0.6148
Cl^−^	RF	18.831	0.8316	0.8589	0.5964
HCO^3−^	GP	0.5056	0.4144	0.1782	0.7668
K^+^	RF	0.0241	0.0166	0.3476	0.6024
pH	RF	0.3531	0.2338	0.0298	0.3906
EC	RF	271.5346	149.3192	0.3013	0.7665
TDS	RF	422.6822	196.7117	0.4578	0.5521

**Table 21:** The most influential input variables for predicting water parameters.

	Input Variable															
Output	HSE	CA	SO	BL	F	RL	UA	WB	AA	HSGA	HSGB	HSGC	HSGD	GPGM	GPGN	GPGT
SAR		3			5	1	4		2							
Na^+^		3			1	4			2		5					
Mg^2+^					1	2	3		5					4		
Ca^2+^		4			1		5		3					2		
SO_4_^2−^		4			2	1	5							3		
Cl^−^	5				1		3				2					4
HCO_3_^−^	5	3			2		4							1		
K^+^	5				3				1		4			2		
pH			1			2					4	5			3	
EC					1	2	5		4						3	
TDS	2				1				3	4				5		

## Data Availability

The data presented in this study are available on request from the corresponding author.

## Appendix A

- **Na parameter**

**Table A1:** Comparative analysis of results of different machine learning models for Na parameter prediction.

Model	RMSE	MAE	MAPE/100	R
Decision Tree	2.1568	**0.7220**	**0.8754**	0.4381
TreeBagger	1.6809	0.7675	1.0412	0.5813
Random Forest	**1.6385**	0.8772	1.3929	**0.6780**
Boosted Trees	1.8603	0.9529	1.5436	0.5024
GP exponential	2.5655	1.0096	1.8659	0.0642
GP Sq.exponential	2.8037	1.1203	2.1615	0.0133
GP matern 3/2	2.7865	1.0860	2.0261	0.0314
GP matern 5/2	2.8302	1.1018	2.0680	0.0240
GP Rat. quadratic	2.8037	1.1203	2.1615	0.0133
GP ARD exponential	3.3385	1.3350	2.8318	−0.0282
GP ARD Sq. exponential	3.6399	1.4212	2.5305	−0.0099
GP ARD matern 3/2	4.2629	1.5668	2.8746	0.0350
GP ARD matern 5/2	4.4450	1.6865	3.0962	0.0170
GP ARD Rat. quadratic	4.2855	1.5028	2.6716	0.0366

- **Mg parameter**

**Table A2:** Comparative analysis of results of different machine learning models for Mg parameter prediction.

Model	RMSE	MAE	MAPE/100	R
Decision Tree	0.6043	0.3313	0.3001	0.4911
TreeBagger	0.4048	0.2641	0.2735	0.7472
Random Forest	**0.4020**	**0.2631**	**0.2717**	**0.7567**
GP exponential	0.6173	0.3524	0.3443	0.4355
GP Sq.exponential	0.6711	0.4076	0.4150	0.3733
GP matern 3/2	0.6532	0.3786	0.3731	0.3915
GP matern 5/2	0.6633	0.3907	0.3875	0.3802
GP Rat. quadratic	0.6711	0.4076	0.4150	0.3733
GP ARD exponential	0.6925	0.4114	0.3927	0.3953
GP ARD Sq. exponential	0.7831	0.4364	0.4129	0.3459
GP ARD matern 3/2	0.6877	0.4295	0.4213	0.4068
GP ARD matern 5/2	0.7180	0.4323	0.4207	0.3371
GP ARD Rat. quadratic	0.7291	0.4207	0.4208	0.3987

- **Ca parameter**

**Table A3:** Comparative analysis of results of different machine learning models for Ca parameter prediction.

Model	RMSE	MAE	MAPE/100	R
Decision Tree	0.7057	0.5504	0.2511	0.6804
TreeBagger	0.5949	0.4642	0.2054	0.7496
Random Forest	**0.5847**	**0.4500**	**0.2007**	**0.7496**
Boosted Trees	0.7730	0.6435	0.2808	0.5093
GP exponential	0.9379	0.5540	0.2225	0.3910
GP Sq.exponential	0.8241	0.5888	0.2566	0.5166
GP matern 3/2	0.7853	0.5538	0.2374	0.5662
GP matern 5/2	0.7989	0.5687	0.2447	0.5505
GP Rat. quadratic	0.8093	0.5755	0.2498	0.5364
GP ARD exponential	0.8347	0.6113	0.2617	0.5626
GP ARD Sq. exponential	0.8156	0.5878	0.2515	0.5497
GP ARD matern 3/2	0.7873	0.5772	0.2391	0.5873
GP ARD matern 5/2	0.7825	0.5809	0.2409	0.5948
GP ARD Rat. quadratic	0.9471	0.6581	0.2822	0.4985

- **SO_4_ parameter**

**Table A4:** Comparative analysis of results of different machine learning models for SO4 parameter prediction.

Model	RMSE	MAE	MAPE/100	R
Decision Tree	0.6585	0.3319	**0.4713**	**0.6249**
TreeBagger	0.5997	0.3228	0.5064	0.5535
Random Forest	**0.5526**	**0.3122**	0.5050	0.6148
Boosted Trees	0.6421	0.4283	0.8503	0.5900
GP exponential	0.8183	0.4002	0.6450	0.3296
GP Sq.exponential	0.9090	0.4751	0.8683	0.1869
GP matern 3/2	0.8811	0.4453	0.7749	0.2489
GP matern 5/2	0.8930	0.4592	0.8144	0.2260
GP Rat. quadratic	0.9060	0.4713	0.8580	0.1918
GP ARD exponential	0.8579	0.4061	0.5984	0.2182
GP ARD Sq. exponential	1.0228	0.5025	0.8036	0.1891
GP ARD matern 3/2	0.9006	0.4476	0.8242	0.3506
GP ARD matern 5/2	0.8340	0.4127	0.7664	0.3954
GP ARD Rat. quadratic	0.8370	0.4631	0.8300	0.3486

- **Cl parameter**

**Table A5:** Comparative analysis of results of different machine learning models for Cl parameter prediction.

Model	RMSE	MAE	MAPE/100	R
Decision Tree	2.4687	0.8348	0.9213	0.4090
TreeBagger	1.9022	0.8556	0.6413	0.5878
Random Forest	**1.8831**	**0.8316**	**0.8589**	**0.5964**
Bosted Trees	2.2544	1.0919	1.3900	0.4431
GP exponential	2.9457	1.1626	1.8895	0.0196
GP Sq.exponential	3.2492	1.2872	2.1291	−0.0253
GP matern 3/2	3.2183	1.2545	2.0769	−0.0135
GP matern 5/2	3.2735	1.2793	2.1214	−0.0177
GP Rat. quadratic	3.2492	1.2871	2.1291	−0.0253
GP ARD exponential	3.8178	1.5359	2.7443	−0.0370
GP ARD Sq. exponential	4.1299	1.5817	2.4557	−0.0557
GP ARD matern 3/2	4.9069	1.8010	2.7176	−0.0523
GP ARD matern 5/2	5.3636	2.0316	3.6072	−0.0620
GP ARD Rat. quadratic	4.1299	1.5817	2.4557	−0.0557

- **HCO_3_ parameter**

**Table A6:** Comparative analysis of results of different machine learning models for HCO3 parameter prediction.

Model	RMSE	MAE	MAPE/100	R
Decision Tree	0.6782	0.5473	0.2228	0.5477
TreeBagger	0.5231	0.4400	0.1875	0.7386
Random Forest	0.5174	0.4252	0.1822	0.7280
GP exponential	**0.5056**	**0.4144**	**0.1782**	**0.7668**
GP Sq.exponential	0.5803	0.4791	0.2006	0.6541
GP matern 3/2	0.5312	0.4309	0.1827	0.7287
GP matern 5/2	0.5437	0.4404	0.1859	0.7109
GP Rat. quadratic	0.5516	0.4473	0.1872	0.6994
GP ARD exponential	0.6389	0.5309	0.2225	0.5902
GP ARD Sq. exponential	0.5596	0.4529	0.1860	0.6829
GP ARD matern 3/2	0.5692	0.4750	0.2001	0.6773
GP ARD matern 5/2	0.5986	0.4949	0.2026	0.6417
GP ARD Rat. quadratic	0.5951	0.4967	0.2070	0.6340

- **K parameter**

**Table A7:** Comparative analysis of results of different machine learning models for K parameter prediction.

Model	RMSE	MAE	MAPE/100	R
Decision Tree	0.0308	0.0201	0.4365	0.3385
TreeBagger	**0.0238**	0.0172	0.3722	0.5919
Random Forest	0.0241	**0.0166**	**0.3476**	**0.6024**
GP exponential	0.0275	0.0181	0.4008	0.4880
GP Sq.exponential	0.0306	0.0205	0.4568	0.3393
GP matern 3/2	0.0292	0.0196	0.4344	0.4168
GP matern 5/2	0.0297	0.0199	0.4434	0.3924
GP Rat. quadratic	0.0299	0.0201	0.4486	0.3769
GP ARD exponential	0.0293	0.0192	0.4225	0.4182
GP ARD Sq. exponential	0.0301	0.0189	0.3988	0.3328
GP ARD matern 3/2	0.0311	0.0207	0.4621	0.3086
GP ARD matern 5/2	0.0307	0.0206	0.4682	0.3449
GP ARD Rat. quadratic	0.0315	0.0209	0.4728	0.3148

- **Ph parameter**

**Table A8:** Comparative analysis of results of different machine learning models for Ph parameter prediction.

Model	RMSE	MAE	MAPE/100	R
Decision Tree	0.3719	0.2457	0.0316	**0.4241**
TreeBagger	0.3558	0.2376	0.0303	0.3380
Random Forest	**0.3531**	**0.2338**	**0.0298**	**0.3906**
Boosted Trees	0.3817	0.2576	0.0330	0.3187
GP exponential	0.4155	0.2586	0.0330	−0.0197
GP Sq.exponential	0.4201	0.2622	0.0335	0.0009
GP matern 3/2	0.4183	0.2573	0.0328	−0.0002
GP matern 5/2	0.4172	0.2560	0.0326	0.0114
GP Rat. quadratic	0.4192	0.2613	0.0334	−0.0247
GP ARD exponential	0.4972	0.2970	0.0381	−0.0636
GP ARD Sq. exponential	0.5655	0.3499	0.0453	−0.0511
GP ARD matern 3/2	0.5025	0.3098	0.0400	0.0272
GP ARD matern 5/2	0.5096	0.3127	0.0403	−0.0118
GP ARD Rat. quadratic	0.4154	0.2497	0.0318	0.1367

- **EC parameter**

**Table A9:** Comparative analysis of results of different machine learning models for EC parameter prediction.

Model	RMSE	MAE	MAPE/100	R
Decision Tree	352.6501	168.7962	0.3289	0.5627
TreeBagger	286.5049	151.5407	**0.2797**	0.7664
Random Forest	**271.5346**	**149.3192**	0.3013	**0.7665**
Bosted Trees	297.9335	170.2860	0.3620	0.6393
GP exponential	432.0779	200.1383	0.4037	0.2465
GP Sq.exponential	474.4095	227.5836	0.4762	0.1730
GP matern 3/2	467.6136	217.1865	0.4402	0.1915
GP matern 5/2	472.1707	221.6132	0.4518	0.1856
GP Rat. quadratic	474.4095	227.5836	0.4762	0.1730
GP ARD exponential	461.6100	216.0258	0.4514	0.2684
GP ARD Sq. exponential	674.4802	269.8017	0.5526	0.1942
GP ARD matern 3/2	631.0788	275.7947	0.5870	0.1756
GP ARD matern 5/2	674.8450	287.7216	0.5782	0.1310
GP ARD Rat. quadratic	470.4831	237.1822	0.4714	0.2560

- **TDS parameter**

**Table A10:** Comparative analysis of results of different machine learning models for TDS parameter prediction.

Model	RMSE	MAE	MAPE/100	R
Decision Tree	509.6578	212.4422	0.5367	0.4610
TreeBagger	422.7209	199.4986	0.4718	**0.5535**
Random Forest	**422.6822**	**196.7117**	**0.4578**	0.5521
Boosted Trees	457.8293	235.2232	0.6106	0.5367
GP exponential	617.8458	**274.5861**	**0.7459**	0.0383
GP Sq.exponential	663.3802	302.3803	0.8247	0.0276
GP matern 3/2	662.2055	297.1371	0.8050	0.0191
GP matern 5/2	666.2775	299.6533	0.8082	0.0207
GP Rat. quadratic	663.3802	302.3803	0.8247	0.0276
GP ARD exponential	765.3184	377.8162	1.1147	−0.0131
GP ARD Sq. exponential	818.4166	408.9820	1.1664	−0.0367
GP ARD matern 3/2	881.9864	460.2564	1.4128	−0.0318
GP ARD matern 5/2	828.6321	416.7358	1.3426	0.0524
GP ARD Rat. quadratic	785.2499	392.9596	1.2369	0.0238
